# Boric Acid Post‐Modified [Al]ZSM‐5 Zeolites: Properties, Acidity, and Ethanol/Ethene Conversion

**DOI:** 10.1002/chem.202503502

**Published:** 2026-02-21

**Authors:** Zheng Li, Daniel Dittmann, Dennis Strassheim, Michael Dyballa

**Affiliations:** ^1^ Institute of Chemical Technology University of Stuttgart Stuttgart Germany

**Keywords:** boric acid modification, ethanol/ethene/ETA conversion, zeolite acidity, ZSM‐5

## Abstract

The effect of boric acid post‐modification on [Al]ZSM‐5 zeolites is investigated. Boric acid modification reduces surface areas, and high loadings lead to agglomeration of crystals. ^11^B MAS NMR spectroscopy indicates boron isomorphously substituted into the framework if 1.5 wt% boric acid is applied, while above 5 wt% loading additionally surface bound ^B^T^1^ species and, increasingly, boric acid deposits ^B^T^0^ are found. The Brønsted acid site (BAS) density decreases with boric acid loading. ^1^H MAS NMR spectroscopy after acetonitrile‐*d*_3_ loading reveals a similar BAS strength as the parent, thus BAS are associated with bridging Si(OH)Al from the parent's structure. A loading with trimethylphosphine oxide (TMPO) is sensitive to weaker Lewis acid sites (LAS). A subsequent hydration changes the nature of water‐accessible boric acid surface species. Conversion of methanol, ethanol, or ethene over boric acid‐modified MFI was tested. For 1.5 wt% loading, an increased BTEX content and lifetime in ethanol conversion was observed, while other modified catalysts were outperformed by the unmodified parent. Most boron is removed after catalytic application. It is concluded that the introduced weak surface acidity and the introduced LAS density could render the modification method interesting for synthesizing new adsorbents operated at moderate conditions.

## Graphical abstract

The modification of MFI zeolites with boric acid leads to the formation of framework boron, unstable surface species, and unbound deposits. Relevant BAS for catalysis and access to pores are strongly reduced. The boric acid is removed upon converting C1 or C2 alcohols and ethene.

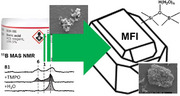

## 1 Introduction

Zeolites are crystalline porous silicoaluminates whose shape‐selective pore system and functional surface groups, as acid sites, enable a broad application in catalytic and separation processes [1]. The zeolite properties, especially the acidity, depend on the amount and location of trace aluminum or other heteroatoms (M) that are incorporated in the zeolite framework [2]. Amongst the possible modifications of zeolites, few have the potential to significantly change the acid site strength of zeolites. The most frequently applied method is the replacement of aluminum atoms with heteroatoms M like gallium, iron, or boron. These heteroatoms have, as a result of their ion size and Lewis acidity, a weaker tendency to form acidic bridging Si(OH)M groups and can in conclusion not in a similar manner generate Brønsted acid sites (BAS) as their aluminum analogue [3, 4, 5]. Common post‐synthetic modifications that alter the acid site density by introduced extra‐framework species are for example phosphatation [6, 7] and partial ion exchange [8]. A less frequently applied method for an acid site modification is the use of extra‐framework boron species after post‐modification with boric acid [9]. Extensive research on this topic was conducted by Sayed and coworkers, whereby it was found that a part of the boric acid were unstable [10, 11, 12, 13]. In particular the BAS density of zeolites was reduced and it was reported that some boron was incorporated into the framework [11]. It is of interest that the reverse route (extraction of boron from the zeolite framework) recently attracted attention for modifying for example zeolite Beta [14]. Removal and reinsertion on the MFI structure was likewise investigated [15]. In both cases, Beta and MFI zeolite structures persist the presence of boric acid without notable damage. Asaftei et al. [16] found activity of boric acid modified ZSM‐5 (MFI structure, 2.56 wt% B) in the conversion of butane/butene mixtures to aromatics (BTX). It was shown that the impregnation to some degree blocks the MFI pores and that B_2_O_3_ forms, which was identified by reflexes in X‐ray diffraction patterns. Also other minerals tend to adsorb large amounts of boric acid in form of deposits, like amorphous aluminum or iron hydroxides, allophane, and kaolinite [17]. Chen et al. [18]. investigated boric acid modified Mordenites in high loadings (2 to 10 wt% boron) by MAS NMR spectroscopy and identified a quadrupolar pattern of trigonal boron at the isotropic shift *δ*_Β,iso_≈ 16 ppm (quadrupolar coupling constant *C*_QCC_ = 2.8 MHz, anisotropy parameter *η *= 0.16). Other peaks were assigned to surface‐bound or framework boron, located at *δ*_Β _= ∼1 to 0.3 ppm (^B^T^2^) and at ‐3.1 ppm (^B^Q^4^). Framework boron species were also identified after boric acid modification of MFI zeolites [11, 12]. Recently it was shown that the isotropic chemical shift of the BO_4_^−^ unit in zeolites correlates with the averaged B‐O‐Si angle [19, 20].

A typical reaction catalyzed by BAS is the conversion of alcohols. Noteworthy, framework boron is not necessarily forming adequate BAS in form of bridging Si(OH)B groups that are strong enough to react with alcohols directly [4, 5]. It is thus worth shortly discussing boron containing, isomorphously substituted ZSM‐5 zeolites as catalysts for the methanol to olefins (MTO) conversion [21]. Pure [B]ZSM‐5 catalyst barely converts methanol [5, 22]. A reactivity of [B]ZSM‐5 in the methanol‐to‐olefin conversion (MTO) is thus usually explained by aluminum impurities [23]. The [B]ZSM‐5 that have been applied in the MTO conversion have reduced BAS density and better crystallinity of the samples, which leads to prolonged MTO lifetime [24]. Similar effects regarding crystallinity and MTO lifetime increase were reported for zeolites of SZR structure [25]. Nevertheless, it was also reported that [Al,B]ZSM‐5 zeolites (Al:B ratio 1:1) performed worse than pure [Al]ZSM‐5 [5]. Thus it is until now unclear if the boron traces in the zeolites have a positive effect themselves [24], or if reported positive effects on the MTO conversion are secondary. The latter means that the incorporated boron improves properties like bulk crystallinity, or accessibility and/or density of BAS [5]. However, no such studies exist for boric acid modified samples and it remains unclear if such a modification with boric acid improves acid‐catalyzed reactions over MFI zeolites [16]. If so, boric acid modification could be a promising alternative to other post‐synthetic modifications like treatment with phosphate. In this work, boric acid modification is applied and changed ZSM‐5 zeolite properties are identified. Therefore, MAS NMR spectroscopy investigations on boric acid modified MFI zeolites are conducted. Acid site nature, density, and strength are for the first time investigated by state‐of‐the‐art probe molecules [26]. Finally, as appropriate test reactions, the modified MFI zeolites are applied in MTO, ethanol‐to‐aromatic (ETA), and ethene conversion and a suited application is discussed.

## 2 Experimental

### 2.1 Sample Preparation

The parent H‐ZSM‐5 zeolite was purchased from Tricat Inc [27]. The modification with boric acid was performed according to Keading [9]. Briefly, 8 g H‐ZSM‐5 were refluxed for 20 h at 353 K in a solution of 0.2 (B1), 0.6 (B2), or 1.25 (B3) g boric acid (Fluka) in 50 mL demin. H_2_O. The water was subsequently evaporated at 353 K and the materials calcined in air for 6 h at 823 K.

### 2.2 Characterization Methods

X‐ray diffraction (XRD) was measured on a Bruker D8 diffractometer using CuKα radiation (λ = 1.5418 Å) in a 2θ range of 3–50°. Crystallinities were calculated by comparing the reflexes of the MFI structure to the amorphous scattering using the Bruker software EVA after subtraction of the instrument background scattering. The chemical composition of the catalysts was determined using an IRIS Advantage ICP‐OES instrument. Scanning electron microscope (SEM) images of the catalysts were collected on a Hitachi CamScan 44 after sputtering the surfaces with gold. N_2_‐physisorption was measured on a Quantachrome Autosorb 3B instrument at 77 K and before the measurement samples were activated for 16 h at 623 K. Surface areas were calculated according to the Brunauer–Emmett–Teller (BET) equation. Mesopore volumes were calculated from the total pore volume at p/p0 = 0.99 minus the micropore volume according to the V–t method (deBoer). Mesopore diameters were assessed via the Barrett–Joyner–Halenda (BJH) method from the adsorption branch. If not stated otherwise, MAS NMR spectra were recorded on a on a Bruker Avance III 400WB spectrometer at 9.4 T in 4 mm rotors and at 8 kHz rotation speed (MAS) and indirectly referenced to TMS. ^1^H MAS NMR measurements were performed on activated samples (treated 12 h at 745 K in vacuum) at a resonance frequency of 400.1 MHz applying π/2 single pulse excitation and with a repetition time of 20 s between scans. For quantitative ^1^H MAS NMR spectroscopy a dehydrated zeolite H,Na‐Y (ammonium exchange degree of 35%) was used as an external standard. The dry samples were treated in a glove box purged with dry N_2_. ^11^B MAS NMR measurements were performed on hydrated or dehydrated material at a resonance frequency of 128.4 MHz upon π/8 single pulse excitation. The probe background was eliminated by subtracting the FID of an empty rotor. ^27^Al MAS NMR measurements were performed on fully hydrated material (measurements on dehydrated material are indicated) at a resonance frequency of 104.2 MHz upon π/12 single pulse excitation. The use of the probe molecules is in detail described elsewhere [26]. Briefly, the dry material was loaded with 70 mbar acetonitrile‐d_3_ (99.9% deuterated, ACROS) via a vacuum line and physisorbed molecules removed in vacuum at 293 K over 12 h. Other samples were loaded with 60 mbar ammonia gas (Westfalen) for 10 min and subsequently evacuated at 453 K for 2 h. Signals of Lewis acid sites (LAS) in the difference spectra were integrated in the range from 5 to ‐0.5 ppm [5]. The liquid loading with trimethylphosphine oxide (TMPO) was conducted according to literature [28]. Briefly, activated and dehydrated catalyst was impregnated with 0.5 mL of a TMPO/CH_2_Cl_2_ solution under N_2_. The concentration of TMPO was calculated as 120 – 140% of the expected BAS density that was previously determined by NH_3_‐loading. It was ensured that the solid was completely covered by the liquid. The solid‐liquid mixture was equilibrated for 30 min, the solvent was removed in flowing N_2_ overnight, and persistent solvent traces were removed over 10 min in vacuum at 323 K. The spectra were processed using Topspin software (Bruker) and DMFit [29].

### 2.3 Catalytic Testing

The catalytic testing was performed as in previous publications [5, 30]. Briefly, all materials were pressed and sieved into a particle size distribution between 200 and 315 µm and then diluted with sea sand (Grüssing, Germany) to a uniform bed height of 5.3 to 5.6 cm in a fixed bed reactor (diameter 7 mm). The herein applied reaction conditions were previously optimized for BTEX formation (*p*_Ethanol_ = 0.3 bar, *T* = 673 K, WHSV = 4 h^−1^). In absence of catalyst no blind conversion occurred. The materials were in situ activated in a nitrogen flow of 50 mL/min by a heat treatment at 383 K for 1 h (3 K/min heating rate) followed by 723 K (1.9 K/min heating rate). After 0.5 h at 723 K the temperature was reduced to the reaction temperature of 673 K. Ethanol was fed into the reactor by a nitrogen flow of 15 mL/min that passed a saturator filled with chromosorb at 326.5 K. All piping after the saturator until the GC inlet was heated to above 393 K. Ethene feed was fed directly into the reactor from a mixture of 40.2% ethene in N_2_ (prepared by Westfalen, Germany) and the WHSV/flow adjusted by dilution with N_2_. The product stream was analyzed using a Hewlett Packard series II 5890 GC equipped with an FID and an Agilent PoraPLOT Q column (52.5 m, 0.32 mm, 10 µm). GC measurements were performed every 53 min and if not stated otherwise the herein shown product distribution was determined after 1 h time‐on‐stream (TOS).

## 3 Results and Discussion

### 3.1 Physicochemical Characterization of Boron‐Zeolites

Physicochemical characterization data of parent and of boric acid (B) post‐modified zeolites B1 to B3 is found in Table 1. The chemical composition of samples determined by chemical analysis reveals a comparable *n*_Si_/*n*_Al_ ratio of 20 throughout the herein investigated samples. As expected, the modification with boric acid is thus not removing the initially present aluminum. The boron content (in wt% dry boric acid per dry mass zeolite) increases in the order B1 to B3 from 1.5 to 10.4%, which equals bulk *n*_Si_/*n*_B_ ratio values of ∼60 to ∼8. As large amounts of boric acid are deposited on surface and within pores (*vide infra*) the mentioned values must not be confused with framework boron contents. The BET surface area calculated from physisorption of N_2_ is close to 400 m^2^/g for parent H‐ZSM‐5 (378 m^2^/g) and B1 (362 m^2^/g) and decreases upon higher boric acid loadings to 307 m^2^/g for B2 and 211 m^2^/g for B3, respectively. Thus, in‐line with previous findings [16], the deposited boric acid blocks some pores of the ZSM‐5 which causes a decreased pore accessibility. Sample B1 shows similar values regarding BET surface area and water loading as the parent. With increased boric acid loading, an up to ∼30 wt% increased water content is monitored for B2 and B3. Note that the higher water content is found despite the inner surface area of B2 and B3 decreased down to a minimum value of 211 m^2^/g. In this respect it is important to note that it was previously reported that adsorbed water mobilizes the boric acid [10]. Thus the question arises if the water is adsorbed or stems from the boric acid itself (and emerges upon condensation of B(OH) groups, as described elsewhere [10]). This question will be answered in the next section.

**TABLE 1:** Physicochemical properties of parent H‐ZSM‐5 and derived samples B1 – B3 after boric acid modification.

Catalyst	*n_Si_*/*n*_Al_‐ratio^a^	*n_Si_*/*n*_B_‐ratio^a^	B(OH)_3_ content [wt%]	BET surface [m^2^/g]	Mesoopore Volume [m^3^/g]	BAS^b^ [mmol/g]	LAS^b^ [mmol/g]	Relative crystallinity^c^ [%]	Water [wt%]^d^
[Al]ZSM‐5	20	∞	0	378	182	0.78	< 0.01	95	9.2
B1	20	59.7	1.5	362	184	0.52	< 0.01	85	9.0
B2	20	16.1	5.4	307	214	0.20	0.05	90	10.4
B3	20	7.7	10.4	211	73	0.10	0.06	85	12.5

^a^ Chemical composition determined by ICP‐OES.

^b^ Quantitative ^1^H MAS NMR spectroscopy, BAS and LAS from ammonia loading.

^c^ From XRD patterns, accuracy ±5.

^d^ Determined by TGA on fully hydrated samples.

**FIGURE 1:**
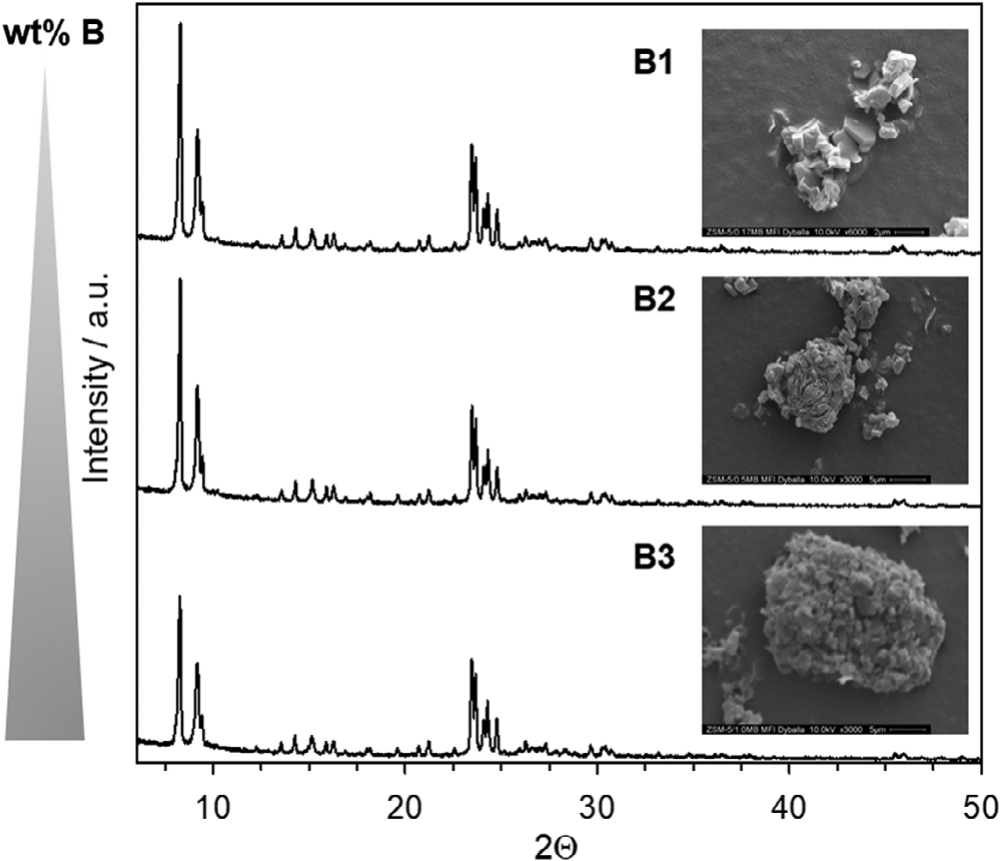
X‐ray diffraction patterns and SEM pictures of ZSM‐5 samples after modification with increasing amounts of boric acid. Magnification of SEM: 6000x, 3000x, 3000x (from top to bottom).

The X‐ray diffraction (XRD) powder patterns of modified zeolites B1 to B3 can be found in Figure 1. All catalysts show the typical reflexes of the MFI topology identical to the parent (see elsewhere) [8]. The patterns are, within the accuracy of the measurement, identical. No reflexes of competing phases are found. Reflexes at 28.10°, according to literature caused by crystalline B_2_O_3_ species, are absent [16]. We also note a decreased crystallinity of boric‐acid modified samples. It is presumably caused by amorphous deposits of boric acid and concluded that these deposits have no long‐range order, presumably due to their noncrystalline, amorphous state. In‐line with literature on boric acid modified MFI and MOR samples [12, 18], also herein the zeolite framework stays widely intact. It is concluded that the formation of an amorphous B_2_O_3_ phase occurs upon boric acid modification (see also ^11^B MAS NMR in the next section). Due to the presumed absence of a binding to the zeolite surface, also a certain instability of the phase, mentioned in previous literature [10, 31], is to be expected. SEM pictures of representative crystal agglomerates are also depicted in Figure 1. The individual MFI crystals visible in case of the parent (see Figure S1) are also found in case of B1, whereas in case of B2 and B3 the individual crystals are agglomerated to larger structures. The agglomeration of crystals increases upon increasing the boron content of the samples. The boric acid thereby acts as weak binder and covers the external surface of zeolite crystals. It can be concluded that the agglomeration caused by boric acid deposition is the reason for the decreased BET surface area of the samples. This is in‐line with previous literature on high boric acid loadings in post‐modified samples [32]. Further insight arises from calculating the mesopore volume and mesopore diameters, respectively (see Figures S2–S5). The mesopore volume is similar for parent and B1, increases for B2, and strongly decreases (along with total pore volume) for sample B3. This indicates, in‐line with SEM images, the formation of a mesopore‐rich “binder” phase. If the boric acid loading is further increased for B3, the pore volume decreases again due to the deposition of boric acid. With increasing boric acid content, we find an increasingly strong hysteresis in the isotherms. Applying the BJH method, we could not find a distinct mesopore diameter. This is typical for inhomogeneous samples having a (meso)pore diameter distribution.

### 3.2 Bulk Material Properties by ^11^B and ^27^Al MAS NMR Spectroscopy

**FIGURE 2:**
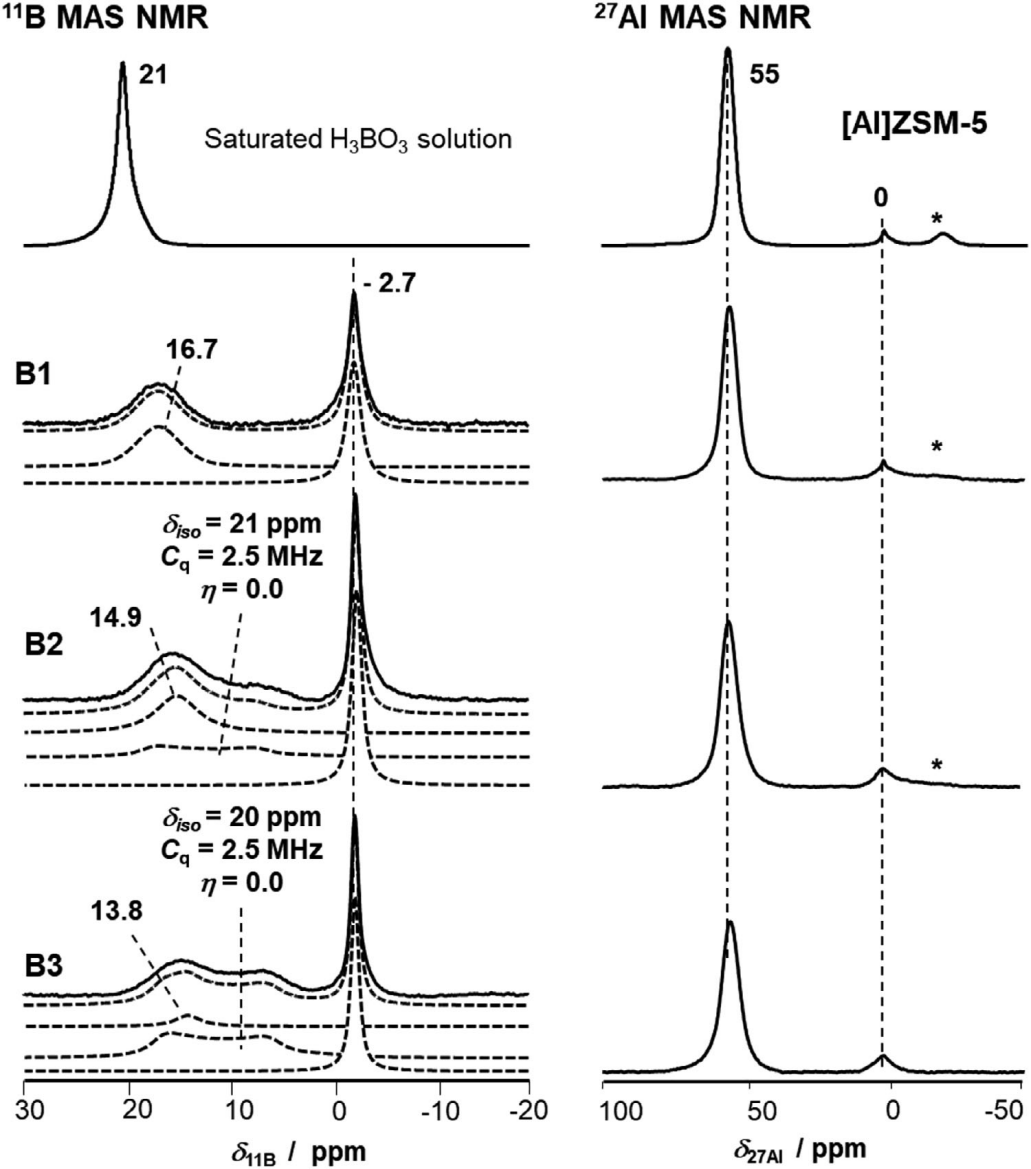
^11^B and ^27^Al MAS NMR spectra of (from left to right and top to bottom) saturated boric acid solution, parent zeolite [Al]ZSM‐5 and boric acid modified B1, B2, and B3, all in their fully hydrated H‐forms. Spinning sidebands are marked by asterisks (*). Fitted spectra are shown as dotted lines and include, from top to bottom, the total fitting spectrum and individual peaks.

The catalytically active BAS of herein investigated zeolites originate from an aluminum doping of the parent framework. In this work, additional boric acid is deposited on the surface of parent [Al]ZSM‐5 zeolites. This could result in additional acidity caused by the introduced boron. Both ^11^B and ^27^Al MAS NMR spectra might give important insights on the acid sites in the boric acid modified samples (see Figure 2). For referencing, the ^11^B MAS NMR spectrum of boric acid in aqueous solution and the ^27^Al MAS NMR spectrum of parent [Al]ZSM‐5 zeolite are also shown on top. To understand the ^11^B MAS NMR spectra, we apply the (silicon‐analogue) nomenclature suggested by Kroeker and Stebbins [33] that was later also supported by Koller et al. [34]. Briefly, tetrahedral framework boron (BO_4_^−^ species, isomorphously substituted boron) is named ^B^Q^4^ whereas trigonal framework Boron is named ^B^T^3^ and free, extra‐framework boric acid with zero surface bindings is named ^B^T^0^. For both ^B^Q^n^ and ^B^T^n^ species, n indicates the amount of dehydroxylated boric acid hydroxyl groups. ^B^T^3^ indicates thus that the boron species forms n = 3 bindings over oxygen to other atoms like, in particular, silicon. The found chemical shifts *δ*_11B_ in ^11^B MAS NMR spectra span a chemical shift range from 21 ppm to about ‐2.7 ppm.

The slim peak of framework boron, in tetrahedral coordination upon hydration of the sample, is herein found at *δ*_11B_ = ‐2.7 ppm. This agrees well with previous literature on isomorphously substituted [B]ZSM‐5 zeolites [35, 36, 37]. The herein found presence of this species in all three boric acid modified samples proves that some boron from the boric acid is isomorphously incorporated into the framework during the post‐modification. This is in‐line with initial findings on boric acid modified MFI [10]. Usually, such species are generated by a direct synthesis of [B]ZSM‐5 catalysts [34]. The ^11^B MAS NMR spectra in dehydrated state (12 h at 745 K in vacuum) are found in Figure S6. They are compared with the spectrum of a directly synthesized [B]ZSM‐5 zeolite (for details on this zeolite refer to previous work [5]). It is immediately observed that the quadrupolar pattern in the spectrum of B1, that covers a chemical shift range *δ*_11B_ from about 10 to‐5 ppm, agrees with that in the spectrum of [B]ZSM‐5. This again supports presence of a ^B^Q^4^ site of boron incorporated into the framework, in‐line with existing literature [38, 39]. The strong quadrupolar interaction emerges upon dehydration of boron‐containing silicates. This induces a transformation of tetrahedral B^4^ into a trigonal coordinated B^3^ species. A likewise behavior was for example observed in Boralite, where a negligible quadrupolar interaction for ^B^Q^4^ species emerges into *C*_QCC_ = 2.55 MHz (*η *= 0.0) for ^B^T^3^ species upon dehydration [35]. In general, trigonal boron species in boranes have usually *C*_QCC_ in between 2.4 to 2.9 MHz [33]. The value agrees also specifically with literature reports on [B]ZSM‐5 showing *C*_QCC_ = 2.7±0.1 MHz (*η *= 0.1) for trigonal ^B^T^3^ compared to *C*_QCC_≤ 0.85 MHz (*η *= 0.0) for tetrahedral ^B^Q^4^ species [40]. The switch from tetrahedral to trigonal framework boron appears for H‐form zeolites. It can be prevented by either ion‐exchange of the Si(OH)B group proton with a stabilizing cation [38] or by inserting a sufficiently strong base coordinating at the acidic proton [5, 41].

An additional peak, with negligible quadrupolar interaction, is found in Figure 2. Its maximum is located at *δ*_11B_ = 16.7 ppm for B1 and shifts to higher field with increasing boron loading, to *δ*_11B_ = 14.9 ppm for B2 and to *δ*_11B_ = 13.8 ppm for B3, respectively. The intensity of the peak decreases thereby. Peaks in this range are usually associated with, to a certain degree, surface‐bound boron species [34, 38]. For example, a ^B^T^2^ species results in chemical shifts *δ*_11B_ of 12.7 to 12.1 ppm in zeolite [B]Beta [42]. In other literature, boric acid modification of Mordenite was suggested to generate a boron ^B^T^2^ site. The respective peak appeared as slim line between 1 and 0.3 ppm with negligible *C*_QCC_ [18]. However, herein no such peak appears. Furthermore, the in [18] reported shift is far off the other literature values of ^B^T^2^ species. We thus conclude the reported species is rather associated with the Mordenite structure or originates from the washing treatment that was performed by the authors after boric acid modification. Chemical shifts *δ*_11B_ of 15.4 to 14.6 ppm were observed on [B]Beta, [B]SSZ‐33, and [B]SSZ‐42 zeolites and assigned to ^B^T^1^ species [3, 43]. Saturated boric acid solution (^B^T^0^) results in a slim peak at ∼21 ppm (see Figure 2). From literature and Figure 2 it becomes clear that the resonance of the boric acid species shifts to lower field upon reducing the binding to the surface. Since the hydration of Si(OH)B sites results in peaks of tetrahedral boron at *δ*_11B_ = ‐2.7 ppm [5], water can be applied for checking the accessibility of framework boron species. Taking into account the above‐made statements, the herein observed chemical shifts *δ*_11B_ of 16.7, 14.9, and 13.8 ppm (for B1, B2, and B3, respectively) are thus assigned to largely surface‐detached ^B^T^1^ species. Furthermore, the presence of quadrupolar patterns in hydrated samples like in Figure 2 is a clear indication of a, for the probe molecule water, not completely accessible inner surface. The well‐accessible B1 sample generated largely detached ^B^T^1^ species, despite the pore surface is completely available for binding boric acid and water can easily access the species. In comparison, on the largely blocked samples B2 and B3, with large deposits of boric acid present, it is unlikely to generate stronger bound ^B^T^2^ species. However, on B2 and B3 it is for steric reasons more difficult for the water to coordinate at the boric acid species. The absence of coordinating water oxygen at these boron sites will result in an upfield shift of the peak. Thus, considerations from the physicochemical properties support an assignment of the peaks at *δ*_11B_ = 16.7, 14.9, and 13.8 ppm to ^B^T^1^ species with, presumably, different amounts of interacting water molecules.

In the ^11^B MAS NMR spectra of dehydrated samples B2 and B3 appears at lower field a complex quadrupolar pattern that is composited of multiple sub‐patterns (see Figure S6). This indicates that multiple species with different quadrupolar interaction have formed upon modification of [Al]ZSM‐5 zeolites with boric acid and after subsequent dehydration. Thus, the boron species on the dehydrated samples are far from being homogeneous. Upon hydration, averaging these sub‐patterns to a single pattern is observed, with chemical shifts of up to *δ*_11B,iso_ = 21 ppm and a quadrupolar coupling constant of *C*_QCC_ = 2.6 MHz (*η *= 0.0). The chemical shift agrees with that of saturated boric acid solution. Thus the assignment of the peak to solvated boric acid or ^B^T^0^ species is reasonable and further supported by literature. On H‐[B]Beta a pattern at *δ*_11B,iso_ = 18.5±0.5 ppm (*C*_QCC_ about 2.6 MHz, *η *= 0.0 or 0.1) was assigned to free B(OH)_3_ with unbound trigonal boron (^B^T^0^) [3, 38]. This agrees with the peak of crystalline boric acid (*δ*_11B,iso_ = 18.8 ppm, *C*_QCC_ = 2.55 MHz, *η *= 0.1) [44]. The modification of zeolite Mordenite with boric acid gave comparable spectra, however, even at only 2wt% loading (according to authors) and despite a subsequent washing step the ^11^B MAS NMR spectrum was completely dominated by a quadrupolar pattern with *δ*_11B,iso_≈ 16 ppm, *C*_QCC_≈ 2.8 MHz (*η *= 0.16) assigned to trigonal boron in H_3_BO_3_ and/or B_2_O_3_ [18]. Also other authors assigned similar quadrupolar patterns to extra‐framework BO_3_‐units [45, 46]. This sheds light on the large water contents herein determined by TGA (see Table 1). The water is not in form of coordinated water molecules, but generated in situ by dehydroxylation of boric acid. The water released upon heating is subsequently detected by TGA [10].

The ^27^Al MAS NMR spectra of parent sample and boric acid modified samples B1 to B3 in hydrated state show two slim and symmetric peaks (see Figure 2). The peaks are attributed to tetrahedral framework aluminum (FAl) at *δ*_27Al_ = 55 ppm and to octahedral extra‐framework aluminum (EFAl) at *δ*_27Al_ = 0 ppm. Upon increasing the amount of boric acid, an increased broadening of the peak at *δ*_27Al_ = 55 ppm is observed. It can be explained by a coverage of the sample surface and the Si(OH)Al groups by boric acid, that disturbs the tetrahedral aluminum coordination slightly upon preventing complete and symmetric hydration. Potential dealumination of the framework and EFAl formation would be spotted in these spectra as increased peak intensity at *δ*_27Al_ = 0 ppm, but such increased intensity is absent here. It could be argued the EFAl were covered by deposits, thus inaccessible to water molecules and conclusively NMR‐silent. However, the stability of the FAL has been proven in former literature. Alumination is therein even applied to replace incorporated boron stoichiometrically in order to generate aluminum‐substituted zeolites of new topology (see in particular the work of Zones and Koller) [34, 38, 47]. This indicates a higher stability of the isomorphously substituted aluminum versus boron in zeolites. In absence of water, the ^27^Al MAS NMR spectra of dehydrated samples B1, B2, and B3 show no peak at *δ*_27Al_ = 55 ppm anymore. Thus, the bridging Si(OH)Al group is not closed and the associated ^27^Al peak broadens out due to the strong quadrupolar interactions. A decreased quadrupolar coupling constant would be expected, if a direct binding between boric acid and Si(OH)Al group, similar to the formation of a surface methoxy species (SMS), would occur. SMS lead to clearly visible peaks in the ^27^Al MAS NMR spectra in the *δ*_27Al_ range between ∼250 to ‐500 ppm on zeolite Y [48] or ∼250 to ‐250 ppm on copper‐loaded Mordenite (see Figure S7; slim central peak at 45 ppm, full width at half maximum ∼7.7 kHz; unpublished data from [49]). Considering the from B1 to B3 increased intensity in the ^27^Al MAS NMR spectra in Figure S1 and taking into account the shape of the peak, a direct binding of boric acid at Si(OH)Al groups in form of ^B^T^1^ species is likely. This supports work of Sayed from 1988, who already proposed such a formation of Si(OB(OH)_2_)Al groups on boric acid modified MFI samples after an intermediate thermal treatment [11]. A drawing of the species in hydrated state is found in the original work.

Summarizing, the presented ^11^B MAS NMR spectra indicate the formation of three different NMR‐visible species upon modification of MFI zeolites with increased amounts of boric acid. Species one is framework‐incorporated ^B^Q^4^ indicated by a peak at *δ*_11B_ = ‐2.7 ppm. Species two is extra‐framework ^B^T^1^ (one Si‐O‐B bond) and causes peaks between 16.7 and 13.8 ppm (the frequency decreases with increased boric acid loading). Species three is completely framework‐detached boric acid deposit, ^B^T^0^, present as amorphous and partially crystalline trigonal boron. The modification with boric acid does not change the nature of aluminum incorporated in the zeolite framework. Finally, upon post‐modification with boric acid no dealumination is observed.

### 3.3 Acid Site Characterization by ^1^H MAS NMR Spectroscopy

**FIGURE 3:**
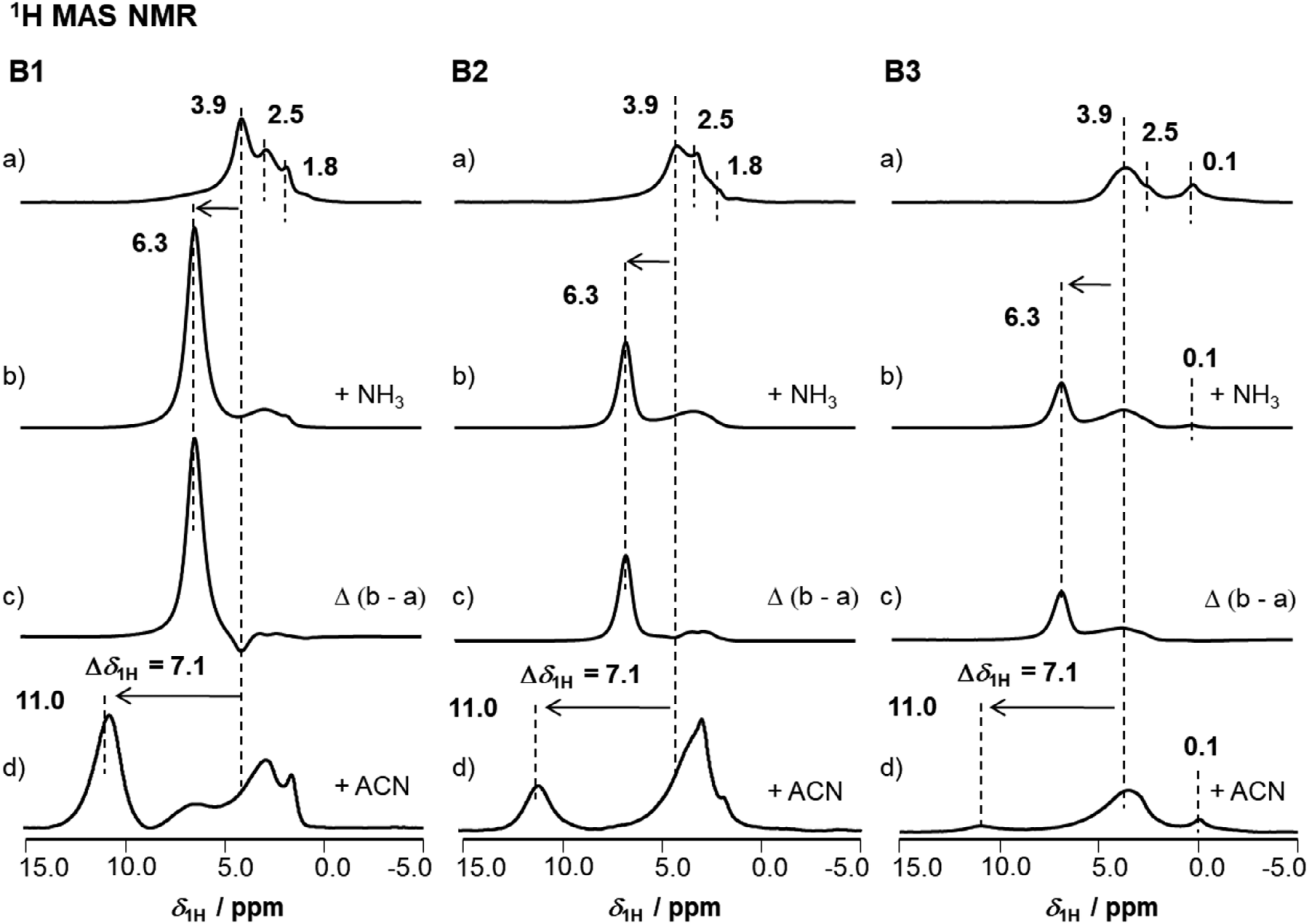
^1^H MAS NMR spectra of boric acid modified ZSM‐5 zeolites after activation (a), loading with ammonia (b) difference spectra for BAS and LAS quantification (c), and acetonitrile‐*d*_3_ (ACN) loading for acid strength evaluation (d).

Zeolite surface hydroxyls can be investigated by ^1^H MAS NMR spectroscopy [2]. In dehydrated state, all samples show the typical peaks of ZSM‐5 zeolites with Si(OH)Al groups at *δ*_1H_ = 3.9 ppm, interacting Si(OH) at *δ*_1H_ = 2.5 – 2.2 ppm, and free Si(OH) groups at *δ*_1H_ = 1.8 ppm [2]. ^1^H MAS NMR peaks of Si(OH) groups interacting with boron species (in form of Si(OH)B groups) and B(OH) groups have been reported in a shift range from *δ*_1H_ = 2.0 to up to 4.3 ppm [34, 38, 50]. A former [51] assignment of a peak at *δ*_1H_ = 0.7 ppm to B(OH) that was correlated with an IR band at 3700 cm^−1^ could herein not be verified. Due to the herein applied activation of the samples, the OH‐groups associated with boric acid have partially condensed and peaks associated with them are largely absent. Thus, no meaningful direct quantification of the Si(OH) density by evaluation of ^1^H MAS NMR peak intensities found in Figure 3a) is possible. In particular on B1 a broad peak around ∼5.5 ppm is caused by the disturbed Si(OH)Al groups of the parent H‐ZSM‐5 (for details refer to [52]). The 3.9 ppm peak intensity decreases with increasing boric acid content in the order B1 to B3, indicating an interaction or coverage of the BAS with boric acid (*vide supra*).

For a more accurate picture on surface hydroxyls, the application of dedicated molecular probes is state of the art [26]. The acid site density of parent and boric acid modified samples was determined by NH_3_ loading and quantitative ^1^H MAS NMR spectroscopy on the formed ammonium ions (see Figure 3b) and results in Table 1). The BAS density decreases upon increasing the amount of boric acid, from an initial density of 0.78 mmol/g for the parent to 0.10 mmol/g for the sample B3. The lowered BAS density upon boric acid modification is explained by blocking of sites [10]. This is in‐line with results from N_2_‐physisorption, SEM, and ^11^B/^27^Al MAS NMR spectroscopy (*vide supra*). The Lewis acidity of the catalysts is quantified via the amount of desorption‐persistent surface‐chemisorbed NH_3_ (see Figure 3c)). Such NH_3_‐species were negligible on the parent catalyst and on B1. Detectable Lewis acid site (LAS) densities were observed for B2 (0.05 mmol/g) and B3 (0.06 mmol/g). This is in‐line with ^11^B MAS NMR spectra for B1 that indicated the boron was isomorphously substituted into the ZSM‐5 framework. Larger amounts of boric acid in case of B2 and B3 lead to deposits. The unsaturated boron deposits give then rise to LAS, comparable to the EFAl that is frequently found in aluminum‐containing zeolite samples. It is important to realize that the boron that was isomorphously substituted into the framework (see ^11^B MAS NMR spectra) is not leading to detectable BAS. The BAS densities in Table 1 thus contain no contribution from Si(OH)B group protons that are too weak to protonate ammonia [5, 40]. This physisorbed ammonia is usually desorbed in vacuum.

The acid site strength of the catalysts was evaluated by loading with a weak base as shifting reagent, here acetonitrile‐*d*_3_ (ACN), and by subsequently measuring the adsorption induced chemical shift Δ*δ*_1H_ of the peak of the Si(OH)Al groups. ^1^H MAS NMR spectra of the samples before and after loading and desorption are found in Figure 3d). Upon loading, the peak of the Si(OH)Al group proton at 3.9 ppm is shifted to lower field. ACN is a shifting reagent which causes a downfield shift of surface hydroxyls in a degree reflecting the hydroxyls’ acid strength [2, 26]. Peaks upfield the BAS peak reflect thus ACN adsorbed at weakly acidic surface hydroxyls, like for example, SI(OH) or here B(OH) groups. For the parent H‐ZSM‐5, an adsorption induced chemical shift of BAS hydroxyls of Δ*δ*_1H_ = 7.1 ppm, as in literature, was measured [52]. Figure 3d) shows that the boric acid modified zeolites B1 to B3 induce exactly the same chemical shift Δ*δ*_1H_ = 7.1 ppm. Thus, the strength of the BAS on parent, B1, B2, and B3 is equal. There is no change in acid site strength upon boric acid modification. In this respect it is important to note that in previous investigations often NH_3_‐TPD was applied to evaluate the acid site strength of boric acid modified catalysts [11, 16]. Thereby the low temperature peak was associated with NH_3_ adsorbed on weak acid sites like Si(OH) or B(OH) groups. Noteworthy this literature conclusion is somehow misleading, as such hydroxyls are no catalytically active BAS in form of Si(OH)Al groups. The formed Si(OH)B groups cannot protonate ammonia and will thus not act catalytically as BAS [5, 40]. In conclusion, boric acid modification does not alter the acid site strength, as verified by the adsorption‐induced chemical shift of the acid proton persisting at Δ*δ*_1H_ = 7.1 ppm. This adsorption induced chemical shift is characteristic for the BAS in pure [Al]ZSM‐5 zeolites. Conclusively the BAS present on the boric acid modified samples are regular Si(OH)Al groups that originate from the parent MFI sample.

### 3.4 ^11^B, ^27^Al, and ^31^P MAS NMR after TMPO Loading and Hydration

**FIGURE 4:**
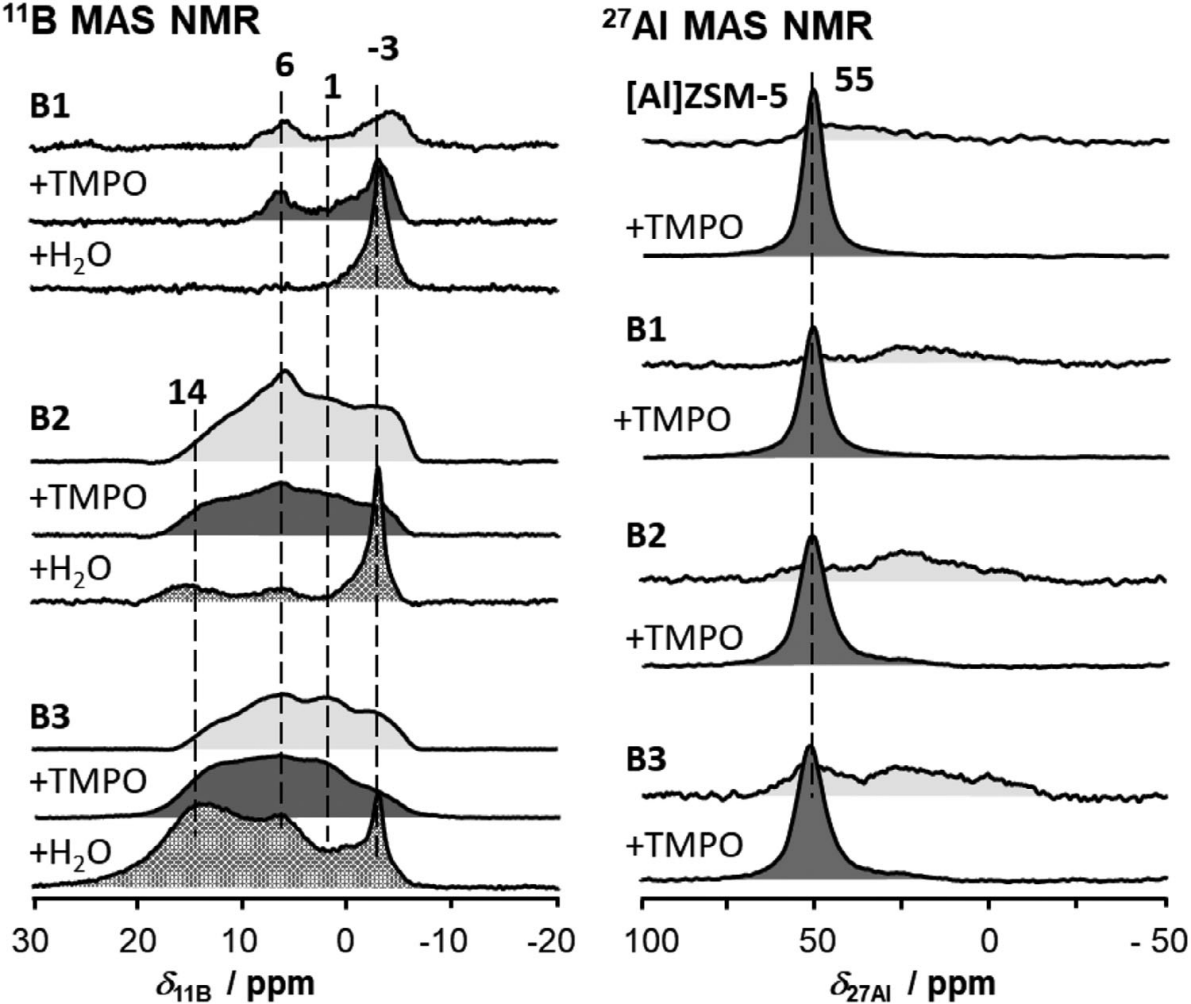
TMPO loading on ZSM‐5 zeolites with boron or aluminum investigated by ^11^B and ^27^Al MAS NMR, respectively. After loading TMPO on dry samples, ^11^B MAS NMR was also measured after subsequent hydration (+H_2_O).

**FIGURE 5:**
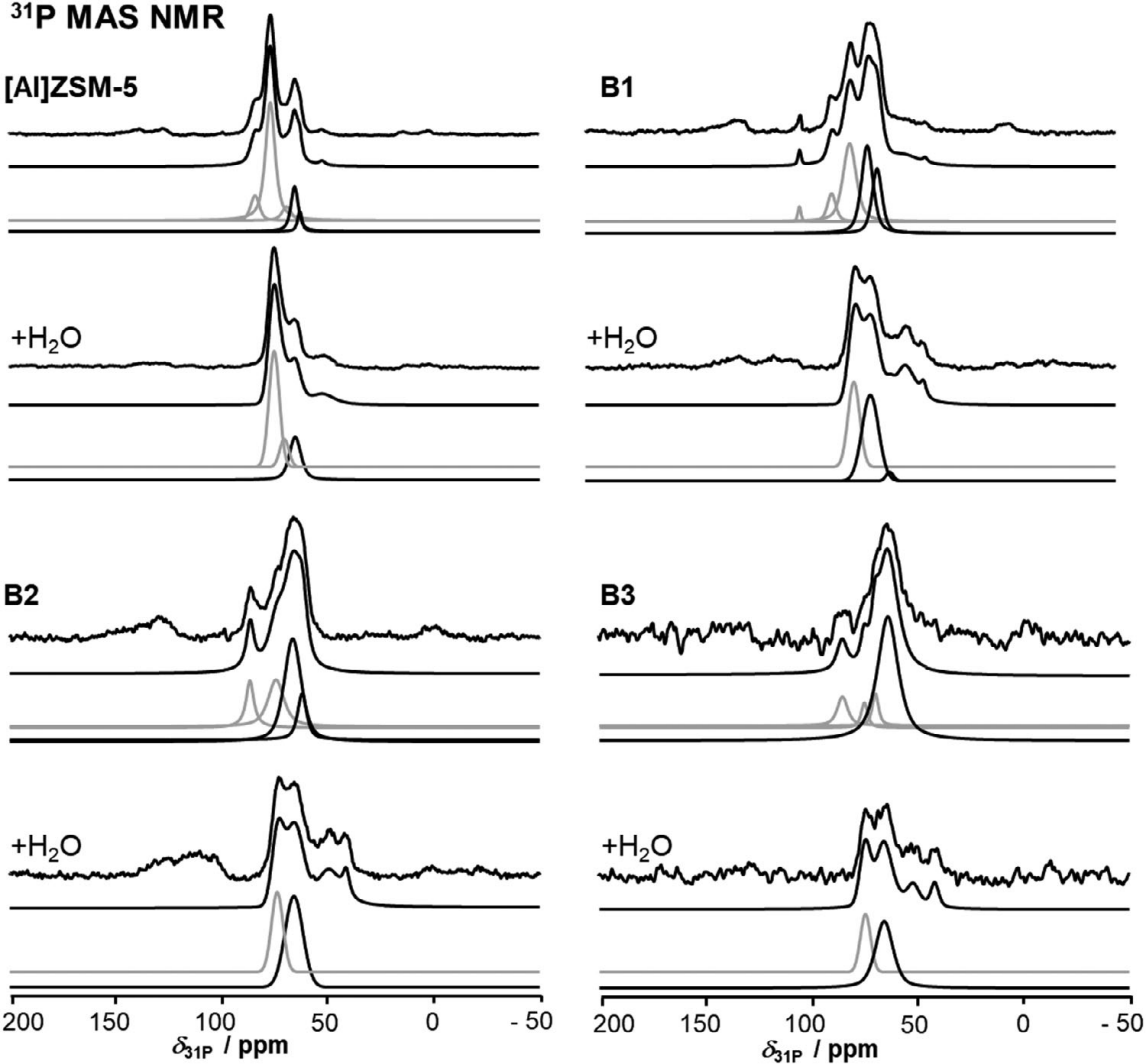
^31^P MAS NMR spectroscopy after loading the dry samples with TMPO (top) and after subsequent hydration of the sample (+H_2_O). Each time original spectra, sum of simulations and peaks assigned to TMPO at Brønsted (grey) and Lewis (black) acid sites are shown (from top to bottom). Physisorbed TMPO is not shown in fittings. All intensities are normalized.

Another broadly applicable probe molecule for acid sites in zeolites is TMPO. TMPO is a comparably strong base, with a proton affinity of 909.7 kJ/mol [53]. This can be considered high compared to other common probe molecules [26]. The TMPO loading is herein followed by ^11^B, ^27^Al, and ^31^P MAS NMR spectroscopy (see Figures 4 and 5). It was proposed that the interaction between TMPO and LAS was weak and that thus water loading of a TMPO‐loaded sample could be useful to distinguish TMPO species on LAS and BAS [50]. This is why herein also a subsequent water loading was applied and included in the investigations.

First a discussion of the ^11^B and ^27^Al MAS NMR spectra of parent ZSM‐5 and of samples B1, B2, and B3 is conducted. The shown spectra correspond, from top to bottom, to activated sample, to activated sample loaded with TMPO, and to the latter after subsequent hydration (see Figure 4). It is immediately observed that upon loading TMPO the ^11^B MAS NMR spectra of samples B1, B2, and B3 barely change. This indicates that TMPO is barely interacting with the present boron species. On B1 and to a minor extent also on B2 a peak at *δ*_11B_≈ ‐3 ppm forms upon TMPO loading. As expected, some of the framework Si(OH)B groups become closed due to an interaction with the sufficiently strong base TMPO [5, 40]. On B3, no indication of a new peak at this position is found. A comparable, incomplete increase in the peak intensity of tetrahedral framework boron upon loading the dry sample with TMPO was recently observed for directly synthesized [B]ZSM‐5 [5]. Conclusively, the interaction of TMPO with Si(OH)B groups is somehow hindered on samples with boric acid deposits. An explanation is the inherent size of the TMPO molecule with a diameter of 0.65 nm that grants access to pores with diameters up to 0.52 nm, as empirically estimated [54]. This is close to the diameter of ZSM‐5 micropores of 0.51 to 0.55 nm and 0.53 to 0.56 nm [55]. Considering the flexibility of the TMPO‐molecule, an access to ZSM‐5 pores is usually possible [54, 56]. But if the pore diameters decrease only slightly, for example due to the deposition of boric acid or due to TMPO deposits/binding, the access of TMPO into ZSM‐5 pores can easily be hindered or prevented. In‐line with these considerations, on samples B2 and B3 the spectra show that upon interaction with TMPO even weaker changes occur than on B1. Simply since on the higher loaded samples most boron is present in form of deposits that block access to the zeolite surface and pores.

Upon hydration, all shown ^11^B MAS NMR spectra change dramatically. An increased peak at *δ*_11B_≈ ‐3 ppm, due to tetrahedral framework boron, is found. First, this implies that the respective groups are far better accessible for the comparably small water molecule (0.33 nm [54]) than for the larger TMPO. The quadrupolar patterns of deposits on samples B2 and B3 also decrease in intensity. However, in particular in the ^11^B MAS NMR spectrum of sample B3, the quadrupolar patterns prevail the hydration, in particular if compared to the spectra of unloaded, hydrated samples (see Figure 4). Again, this is an indication that the samples are less accessible to water. This is reasonable, as the solid TMPO adds up to the already present boric acid deposits and blocks the access. Considering the appearance of new ^11^B MAS NMR peaks in the range between ^B^T^1^ and ^B^Q^4^ species, dramatic changes on the surface must have occurred upon hydration after a TMPO loading. The weak acidity of B(OH) hydroxyl groups would presumably make a binding between them and TMPO unstable (compare absence of binding of TMPO to the stronger acid COOH [57]). With respect to the intensity gain in ^11^B MAS NMR spectra in the *δ*_11B_ range from 20 to ∼0 ppm it can be excluded that TMPO alone is responsible for the new peaks, simply because only few TMPO molecules could access the pores and interact with boric acid deposits prior hydration. It seems upon hydration the basic probe molecule TMPO rather catalyzes condensation reactions between boric acid and surface Si(OH) groups. The higher intensity in ^11^B spectra is caused by the reaction between boric acid and MFI surface instead of between boric acid and TMPO. This leads to the formation of new species (^B^T^2^, ^B^T^3^, and ^B^Q^3^) on the MFI surface. These species were not to be found on the original boric acid modified samples B1 to B3. The TMPO loading and subsequent hydration modified the nature of boron species present on the boric acid modified samples. This indicates clearly that boric acid surface species are unstable under aqueous, alkaline conditions. This however makes a hydration after TMPO‐loading unsuited for a study of the original samples’ surface species.

A different picture after loading TMPO is observed in the ^27^Al MAS NMR spectra. All spectra show an appearance of a peak at *δ*_27Al_ =  55 ppm that is commonly associated with tetrahedral framework aluminum (*vide supra*). This is in‐line with the previous findings on [Al]ZSM‐5 and [B]ZSM‐5 zeolites loaded with TMPO, where likewise interaction between zeolite BAS and TMPO lead to the appearance of the peak associated with tetrahedral framework aluminum [5]. Note that only the aluminum accessible to TMPO will contribute to this peak. In particular, it must be realized that the shown spectra were not taken on hydrated samples. Exclusively the adsorption of TMPO lead to the strong peak *δ*_27Al_ =  55 ppm and the for TMPO not accessible Si(OH)Al groups remain thus NMR‐silent [58]. TMPO is also on boric acid modified samples protonated at Si(OH)Al groups but shows weak interaction with Si(OH)B groups. Barely an interaction with the boron species deposited on the samples is found. Thus, both ^11^B and ^27^Al MAS NMR spectra after loading TMPO and subsequent hydration support the finding from ^1^H MAS NMR after loading acetonitrile‐*d*_3_. Namely, that the BAS present on the boric‐acid modified samples B1, B2, and B3 are those originating from the parent [Al]ZSM‐5. No additional, strong BAS were generated upon boric acid modification.

The next step in the characterization of the samples is the evaluation of the ^31^P MAS NMR spectra collected after TMPO loading and after subsequent hydration. The respective spectra of loaded parent [Al]ZSM‐5, B1, B2, and B3 are shown in Figure 5. The peak assignment to TMPO binding to BAS and LAS (at a *δ*_31P_ of > 66 ppm or 65 to 55 ppm, respectively) as well as to physisorbed TMPO species (around *δ*_31P_ = 50 ppm) is taken from literature [26] and from recent shifts monitored on [B]ZSM‐5 zeolites [5]. Note generally a *δ*_31P_ shift inaccuracy arising from phasing and deconvolution of at least ±1 ppm. Note also that TMPO is not well suited for quantification since the stoichiometry between the probe and the surface sites changes with loading [59]. The resulting semi‐quantitative evaluation of the ^31^P MAS NMR peak intensities, assigned to the two acid sites before and after hydration (+H_2_O) respectively, is found in Table 2. It is immediately realized that the evaluation of BAS and LAS from spectra collected after TMPO loading follows roughly the trends extracted from ^1^H MAS NMR after ammonia loading. They indicate a strong decrease of the BAS density upon modification with increasing amounts of boric acid. The absolute values determined by TMPO are about 50% smaller than these determined after ammonia loading. This is rationalized by the larger diameter of TMPO compared to ammonia [54]. Upon hydration, the peak intensity ascribed to TMPO at BAS decreases. Herein, the BAS are Si(OH)Al groups of similar nature and strength (see ACN loading). It is thus not reasonable to interpret the decreased intensities, as previously suggested, in terms of strong or weak BAS and/or LAS [50]. More probable is a partial hydration and removal of accessible TMPO molecules.

**TABLE 2:** Semi‐quantitative acid site densities after loading TMPO and after subsequent hydration (+H_2_O), all given amounts in mmol/g.

Sample	BAS >66 ppm	(+H_2_O) BAS >66 ppm	LAS 65‐55 ppm	(+H_2_O) LAS 65‐55 ppm
[Al]ZSM‐5	0.78	0.61	0.22	0.26
B1	0.27	0.18	0.35	0.28
B2	0.12	0.07	0.21	0.12
B3	0.04	0.05	0.17	0.12

The semi‐quantitative evaluation of LAS from spectra after TMPO‐loading is less straightforward. By TMPO determined LAS densities are higher than these determined by ammonia. This can be rationalized by the ammonia desorption step that leaves only ammonia at strong LAS behind. This reduced thus the total LAS amount to be quantified. As such a step is absent after TMPO loading, more LAS are found. These LAS might be so weak that they barely interfere with some reactants. It is literature‐known that TMPO dimers at BAS lead to peak intensity in the LAS range, which adds further uncertainty to the tabulated quantitative results [59]. In contrast to ammonia loadings, the TMPO loading indicates thus a maximized LAS density for sample B1. When considering the higher proton affinity of TMPO and the formation of well‐accessible ^B^T^1^ species on the surface of B1, the result is reasonable. An initially surprising finding is a, from B1 to B3, decreasing LAS density, conversely with the increasing boric acid loading. However, if again a reduced access of the TMPO to the LAS in particular on samples B2 and B3 is considered, the results are reasonable (*vide supra*). In particular both physisorption and probe molecule loadings have indicated a decreased inner surface area and a hindered or prevented access to the pore system of the samples. The higher proton affinity of TMPO causes thus the higher LAS density value, compared with ^1^H MAS NMR investigations after ammonia loading. In conclusion, the application of TMPO enables a detection of weaker LAS. Apart from a catalytic application, the found LAS could be of interest for adsorption of molecules.

After hydration the LAS density of all samples decreases. However, as for BAS, the relative amounts agree with the ratios before hydration. If the ^11^B MAS NMR spectra of TMPO‐loaded samples before and after hydration are considered, it becomes clear that the introduced water preferentially interacts with the surface and the sites found there, and to a smaller degree with the TMPO molecules. This is reasonable, considering the TMPO is shielded from interaction with water by methyl groups. In contrast, the zeolite surface is covered with polar hydroxyl groups and boric acid deposits that interact far better with the polar hydroxyls of water molecules. A removal of TMPO from previous adsorption sites is thus rather a function of the TMPO location, the available space around the TMPO, and the thereby changed accessibility for water. It is at least to a smaller extent an indication of the TMPO‐LAS binding strength. It is concluded that a hydration of previously TMPO‐loaded samples results in a misleading picture of the acidity and surface species. This conclusion is in‐line with TMPO‐loadings on [B]ZSM‐5 zeolites [5]. Again, the unclear stoichiometry between probe and site and the often rather arbitrary peak assignment of TMPO‐associated ^31^P MAS NMR peaks renders studies with this probe debatable. But it is now verified that a hydration is not leading to insights on TMPO‐site binding strengths but rather on the water accessibility of by TMPO blocked BAS or LAS.

### 3.5 Catalytic Studies

The boric acid modified catalysts were applied in ethanol‐to‐aromatic (ETA) and ethene conversion under conditions suited for the preferred formation of aromatics [60]. The results are summarized in Table 3, and the respective product distributions over time are found in Figures S8 to S15 in the SI. Supplementary results from MTO conversion and the exemplary propene selectivity are shown in Figure S16.

**TABLE 3:** Selectivity and lifetimes of parent and samples applied in the conversion of ethanol and ethene over H‐form MFI catalysts at WHSV = 4.0 h^−1^, T = 673 K after 1 h TOS.

	C_1_‐C_4_ paraffins [%]	Ethene [%]	Propene [%]	C_4_ olefins [%]	C_5+_ [%]	BTEX aromatics [%]	Lifetime [h]
Ethanol							
[Al]ZSM‐5	20.0	6.4	7.7	15.9	35.0	15.0	17.0
B1	28.0	2.9	5.2	12.3	28.2	23.3	22.3
B2^a^	35.6	1.6	2.0	9.0	23.6	28.1	–^a^
B3^a^	18.9	7.9	8.8	17.2	28.9	18.3	–^a^
Ethene							
[Al]ZSM‐5	27.3	3.6	4.8	11.9	29.4	23.1	29.4
B1	28.5	2.7	4.8	12.1	29.1	22.9	43.5
B2	8.7	38.2	9.6	16.2	24.5	2.8	21.4
B3	10.1	87.1	0.8	1.0	1.0	0.1	0.0

^a^ Values not trustworthy. The removal of boric acid and blocked piping impacted catalytic results.

The shown data is compared to a literature‐known ZSM‐5 catalyst with Si/Al = 29 and BAS density of 0.48 mmol/g [60]. The literature catalyst shows the following selectivity upon conversion of ethanol under identical conditions at WHSV = 4 h^−1^: C_1_‐C_4_ paraffins 16.2%, ethene 9.7%, propene 9.7%, C_4_ olefin 17.8%, C_5+_ 35% and BTEX 11.5%. These are comparable to the values found herein for the parent [Al]ZSM‐5 in Table 1. A higher paraffin and BTEX selectivity is arising from the higher BAS density herein (0.78 mmol/g) which is in‐line with literature (see [60, 61]). The catalytic data of ethanol conversion shows furthermore good agreement with other catalysts measured at WHSV = 3h^−1^ [30, 60, 62], considering again the literature‐known effect arising from a modified WHSV and BAS density [60, 61]. Particular in the ETA conversion over catalyst B1, the modification with boric acid leads to an increased lifetime and increased selectivity to the formation of aromatics and paraffins. This changed selectivity is explained by hindered diffusion in the pores (= longer residence time) and, in particular for B2 and B3, by the presence of strong LAS detectable by ammonia. It is important to mention that the boric acid was washed away from all samples during catalysis and deposits blocked for B2 and B3 the piping after the reactor. Thus, questionable values in Table 1 are flagged as not trustworthy. In‐line with these findings, the boron content of all samples decreased significantly after reaction, as verified by ICP‐OES (see Table S1 in the SI). A similar instability of boron species was formerly reported by Sayed [10] after MTO conversion over boric acid modified MFI zeolites, which decreased selectivity to short olefins for samples B1 to B3.

The by‐product of the reaction, water, was in literature [10] named as reason of the boric acid removal. Thus the conversion of the water‐free feedstock ethene was herein also tested. Results of ethene conversion are also found in Table 3 and a similar removal of boric acid as for ethanol conversion occurred (see Table S1). Despite an initial increase in lifetime is observed for sample B1, most catalytic results, in particular selectivity to aromatics and paraffins, is barely affected. It is noted that it is known that a reduced BAS density leads to an increase in selectivity to propene and C_4_ olefins, as it is herein observed for B1 compared to the parent [60]. In higher boric acid loadings the decreasing BAS density causes a smaller initial conversion and decreases the lifetime. In particular no increased BTEX aromatic content is observed, while for B2 compared to B1 a strongly increased share of propene and C_4_ olefins in the reaction products is found. In parallel the share of paraffins and longer hydrocarbons (C_5+_) decreases. Note that the tabulated ethene is strictly spoken no product of the conversion but the initial reactant, as ethanol is immediately dehydroxylated in the reactor. A similar picture is found for B1, B2, and B3 applied in the MTO conversion. As these results are a repetition of literature‐known work [10], the associated discussion is found along catalytic data below Figure S16. Summarizing, boron species generated upon boric acid modification are unstable under catalytic conditions at elevated temperatures, independent of a water presence. The post‐modification with boric acid does thus not lead to suitable catalysts for hydrocarbon conversion reactions at elevated temperatures. As the modification introduced LAS of different strength, an application of boric acid modified [Al]ZSM‐5 zeolites as adsorptive at ambient conditions seems as conclusion more suited then a catalytic application.

## 4 Conclusions

The post‐modification of [Al]ZSM‐5 zeolites with boric acid was conducted in three increased loading levels. In high boric acid loadings an agglomeration of crystals and reduced surface area was found. ^11^B and ^27^Al MAS NMR spectra of the bulk samples indicate the formation of framework ^B^Q^4^ species, of surface‐bound ^B^T^1^ species, and of extra‐framework boric acid deposits ^B^T^0^. The characteristic ^B^T^0^ deposits’ ^11^B tensor is not transferred into a slim peak after hydration of B2 or B3. A direct condensation between Brønsted acid sites (BAS) and boric acid to ^B^T^1^ species on the BAS is proposed. No dealumination occurred and the parents’ bridging Si(OH)Al groups persisted the post‐modification with boric acid. Quantitative ^1^H MAS NMR after ammonia loading indicates a higher Lewis acid site (LAS) density and smaller BAS density with increased boric acid loading. Loadings with acetonitrile‐*d*_3_ and with TMPO indicate that the present BAS are of same strength and nature as the initial bridging Si(OH)Al sites of the parent. No new BAS are generated upon boric acid modification and detected BAS stem exclusively from the parent [Al]ZSM‐5 framework. After modification with boric acid, new strong LAS were detected by ammonia and TMPO loading. The semi‐quantitative results after TMPO loading enable also the detection of weaker LAS. The inherently large TMPO could thereby not access all surface sites as its access to pores was prevented by boric acid deposits. A subsequent hydration after TMPO loading lead to condensation between surface Si(OH) and B(OH) groups. Thus hydration after TMPO loading modified the nature of boron species on the surface. The boric acid modified [Al]ZSM‐5 performance was evaluated in methanol, ethanol, and ethene conversion reactions. In low boric acid loading, compared with the parent, an increased BTEX formation from ethanol at elevated lifetime was observed. When applying ethene as feed the monitored lifetimes were significantly longer compared to ethanol feed. In particular at larger boric acid loadings of 5 wt% and above, the lifetimes decreased strongly. Similar decreases in lifetime and selectivity to key products were observed in converting methanol. Independent of water formation the boric acid was unstable and largely removed from the surface. All in all the parent usually outperformed the boric acid modified samples in terms of conversion and selectivity. Thus, despite interesting as surface modification route to new adsorbents, the boric acid modification of MFI zeolites is unsuited for catalysts applied at elevated temperatures due to weakly bound and unstable boric acid deposits. Nevertheless, generated weak acidity and LAS could render the material interesting as adsorbent operated under moderate conditions.

## Conflicts of Interest

The authors declare no conflict of interests.

## Supporting information



Supplementary data associated with this article can be found in the online version, at http://dx.doi.
