# Supplementary material for: Boric Acid Post‐Modified [Al]ZSM‐5 Zeolites: Properties, Acidity, and Ethanol/Ethene Conversion
Source: Chemistry. 2026 Feb 21;32(17):e03502. doi: 10.1002/chem.202503502 (PMC13150057; doi:10.1002/chem.202503502)
Supplement: Supplementary file 1 — Supplementary data associated with this article can be found in the online version, at http://dx.doi. [file CHEM-32-e03502-s001.pdf]

Supporting Information (SI)

Boric Acid Post-modified MFI Zeolite: Properties, Acidity and Ethanol/Ethene Conversion

Zheng Li,[1,[a]] Daniel Dittmann,[1,[a]] Dennis Strassheim,[a] and Michael Dyballa[a],*

[a] Institute of Chemical Technology, University of Stuttgart, 70569 Stuttgart, Germany

[1]Both authors contributed equally

*Corresponding author, E-mail: michael.dyballa@itc.uni-stuttgart.de

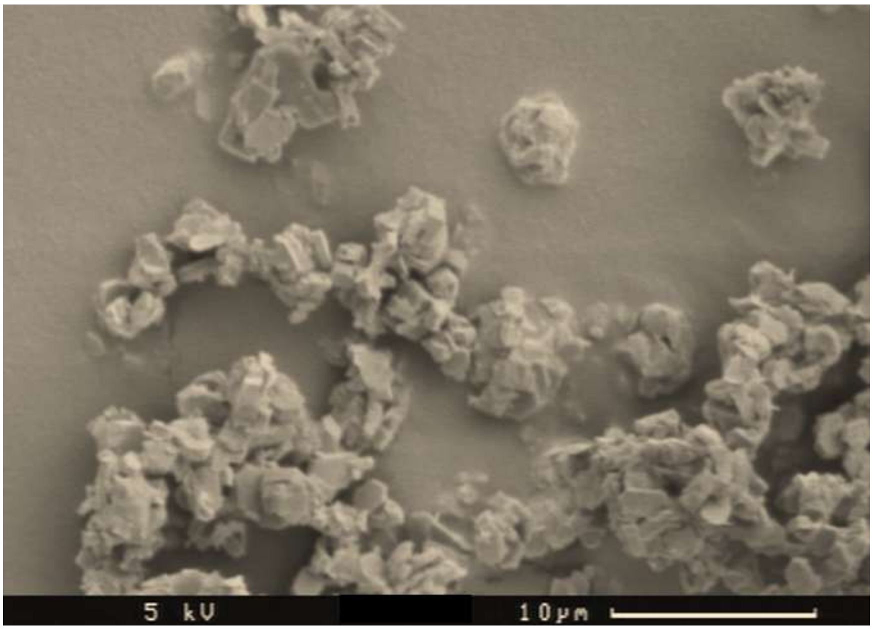

Figure S1: SEM image of the parent [Al]ZSM-5 in a magnification of 4500x. Individual MFI crystals in characteristic coffin-like shape are easily spotted.

On the following pages:

Figures S2 to S5 containing N_2-Physisorption data and evaluation on the samples (in the order parent [Al]ZSM-5, B1, B2, B3).

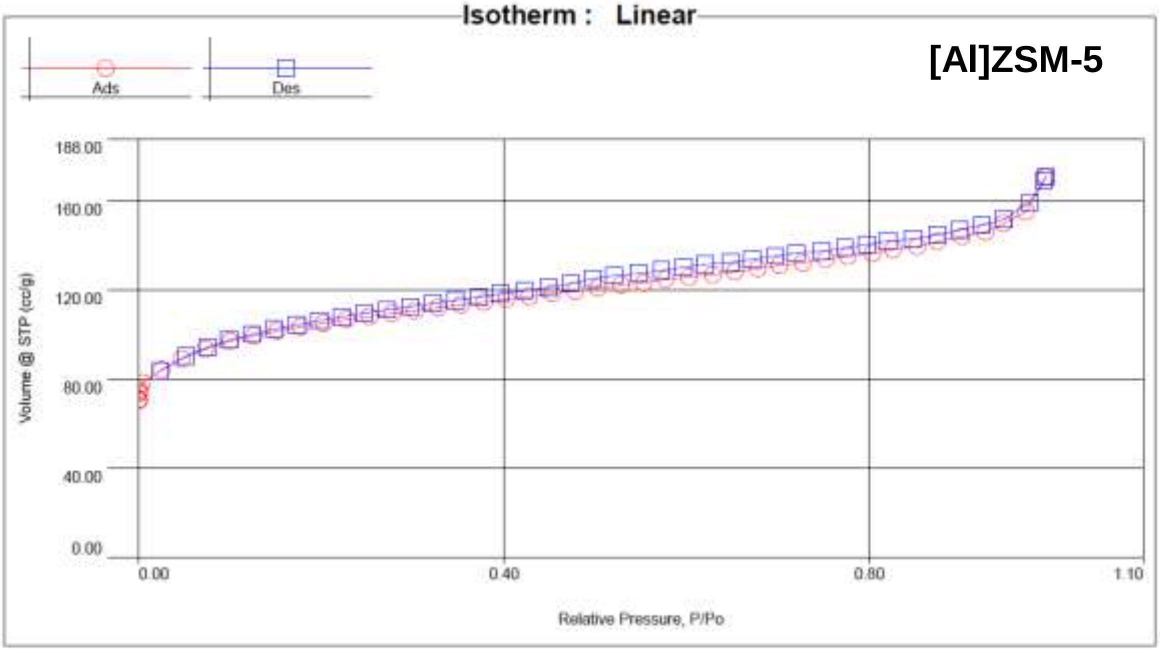

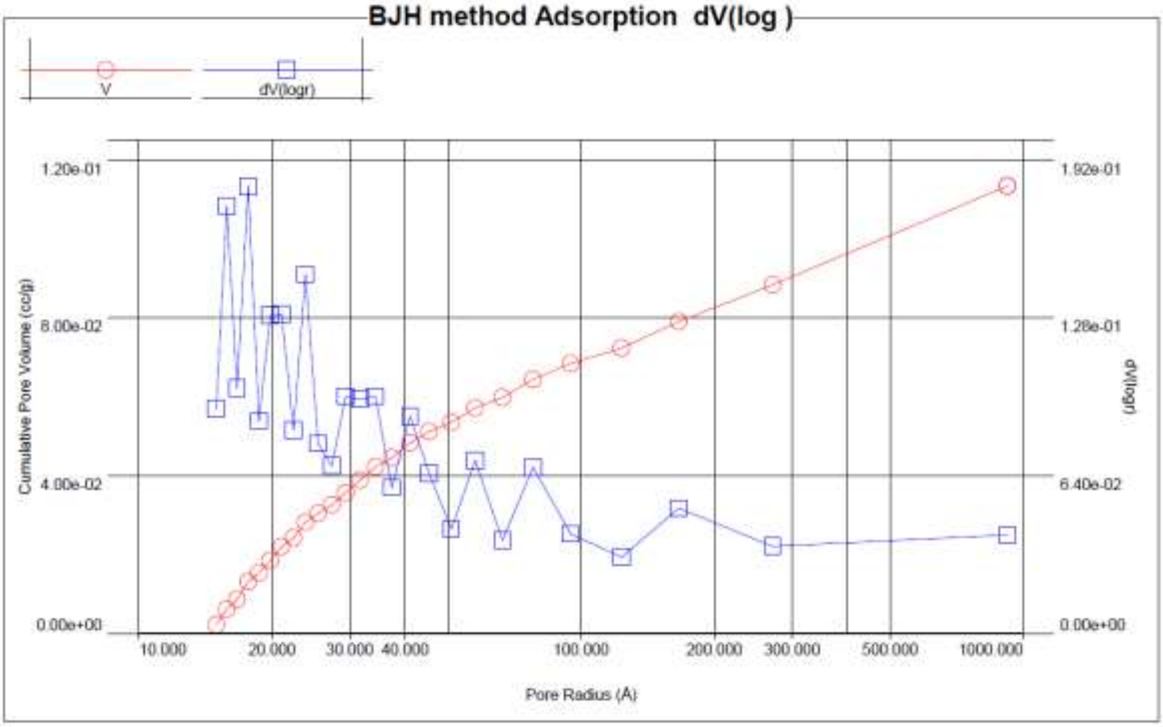

V-t method summary

Thickness method: DeBoer

**Slope =**	11.586
**Intercept =**	53.832
**Correlation coefficient, r =**	0.991163
**Micropore volume =**	0.083 cc/g
**Micropore area =**	198.415 m^2/g
**External surface area =**	179.659 m^2/g

Total Pore Volume data

Total Pore Volume

Total pore volume = 2.649e-01 cc/g for
pores smaller than 1492.0 Å (Radius)
at P/Po = 0.99352

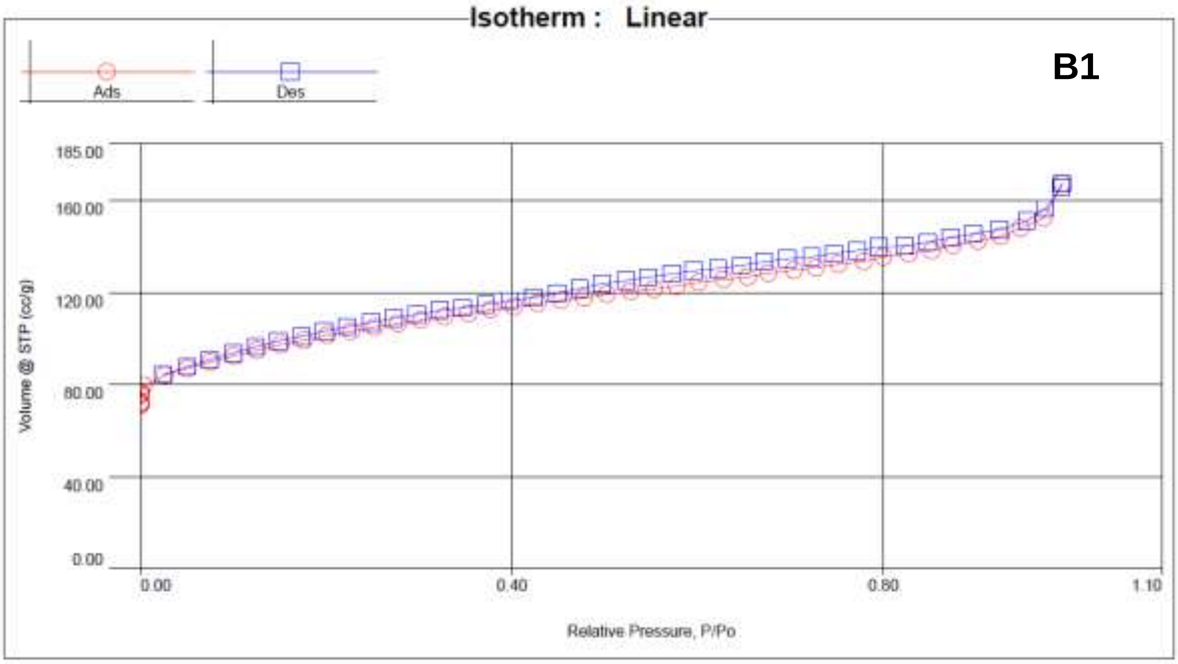

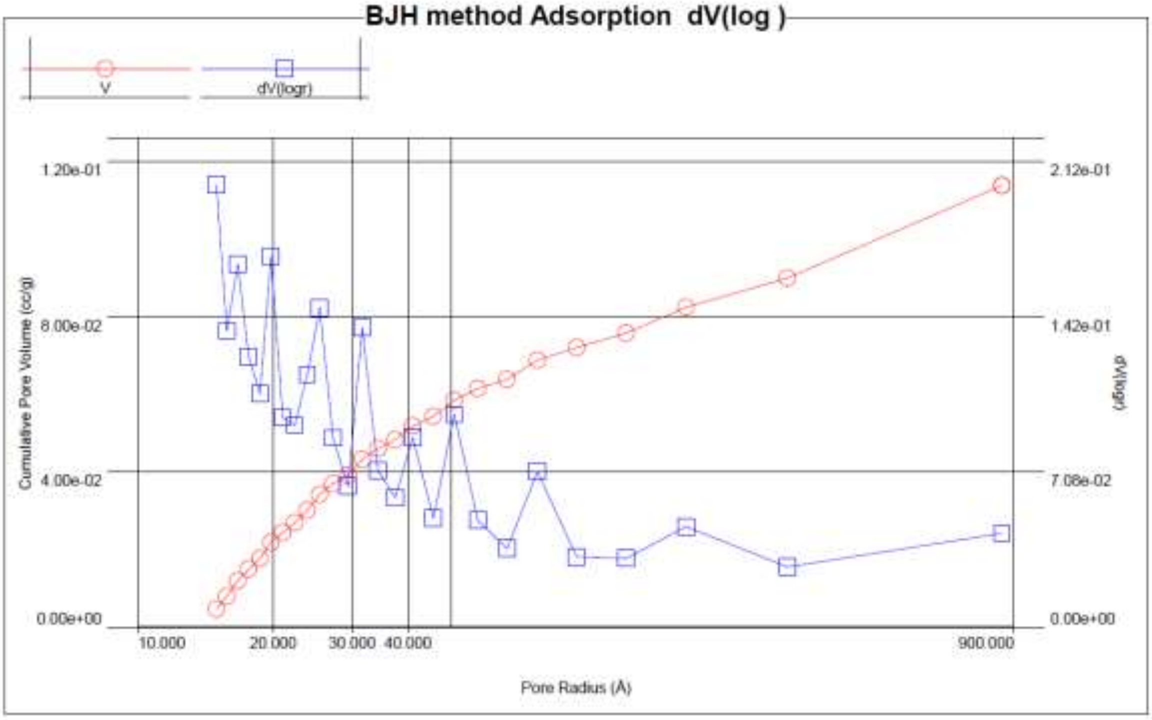

V-t method summary

Thickness method: DeBoer

**Slope =**	12.050
**Intercept =**	48.291
**Correlation coefficient, r =**	0.998383
**Micropore volume =**	0.075 cc/g
**Micropore area =**	175.793 m²/g
**External surface area =**	186.860 m²/g

Total Pore Volume data

Total Pore Volume

Total pore volume = 2.594e-01 cc/g for
pores smaller than 1325.8 Å (Radius)
at P/Po = 0.99270

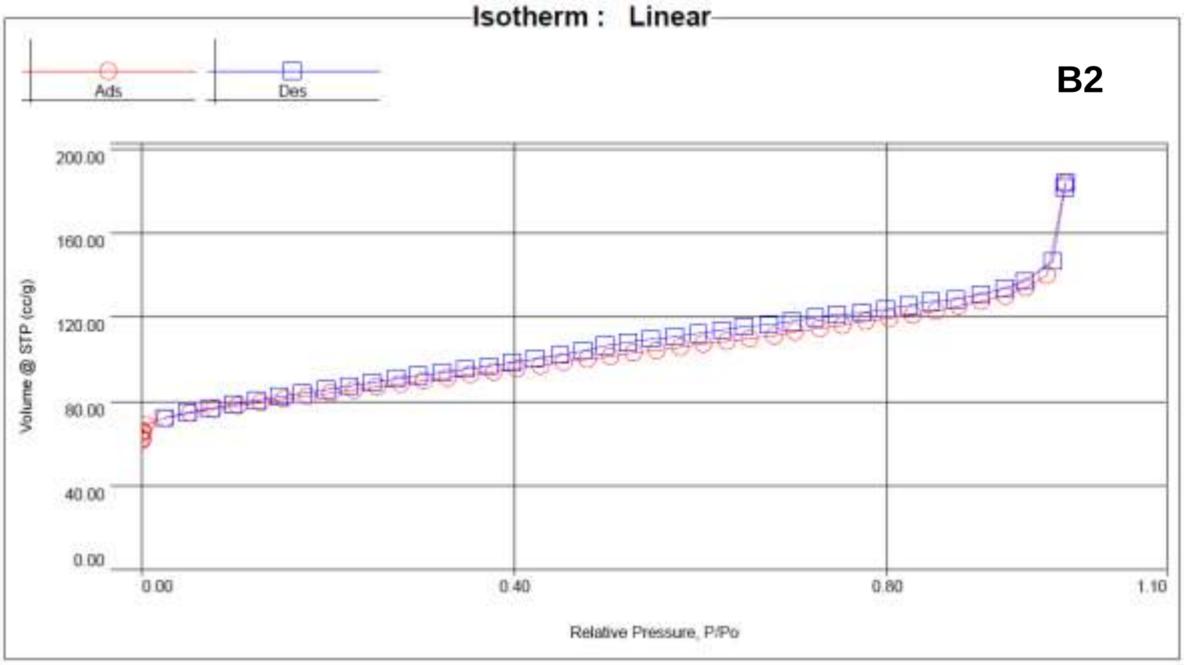

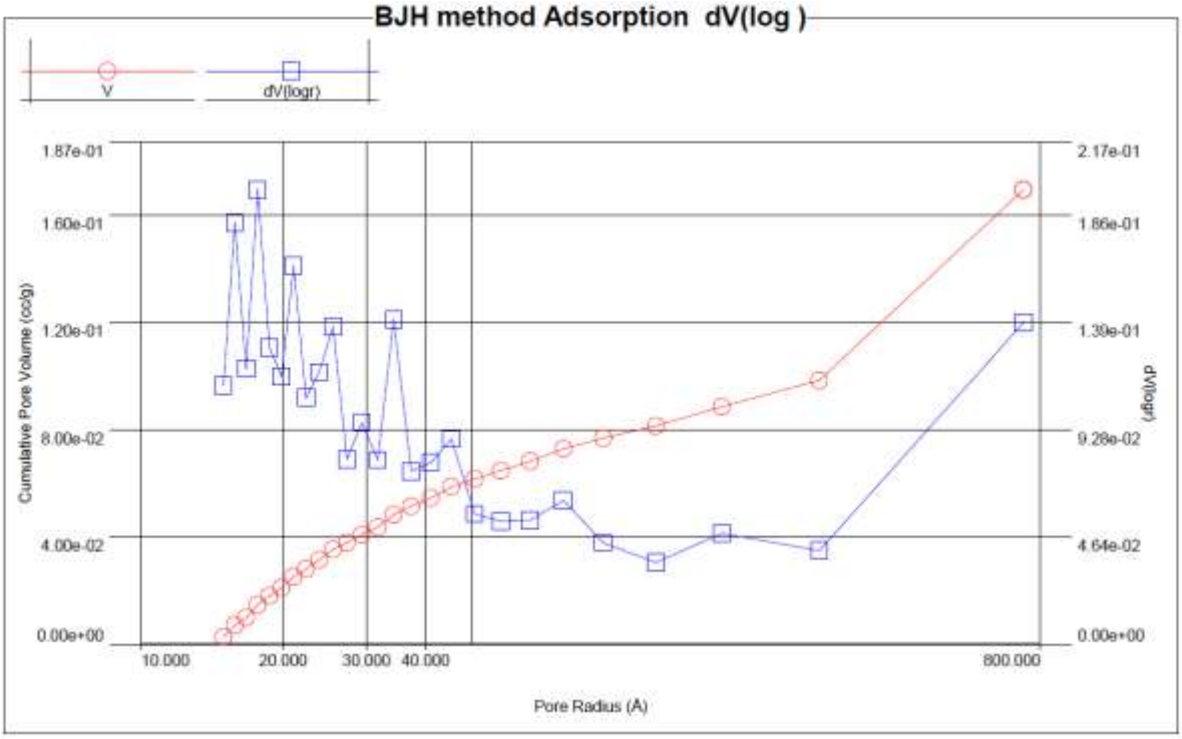

V-t method summary

Thickness method: DeBoer

**Slope =**	8.730
**Intercept =**	45.860
**Correlation coefficient, r =**	0.999559
**Micropore volume =**	0.071 cc/g
**Micropore area =**	171.845 m^2/g
**External surface area =**	135.373 m^2/g

Total Pore Volume data

Total Pore Volume

Total pore volume = 2.850e-01 cc/g for
pores smaller than 1125.8 Å (Radius)
at P/Po = 0.99139

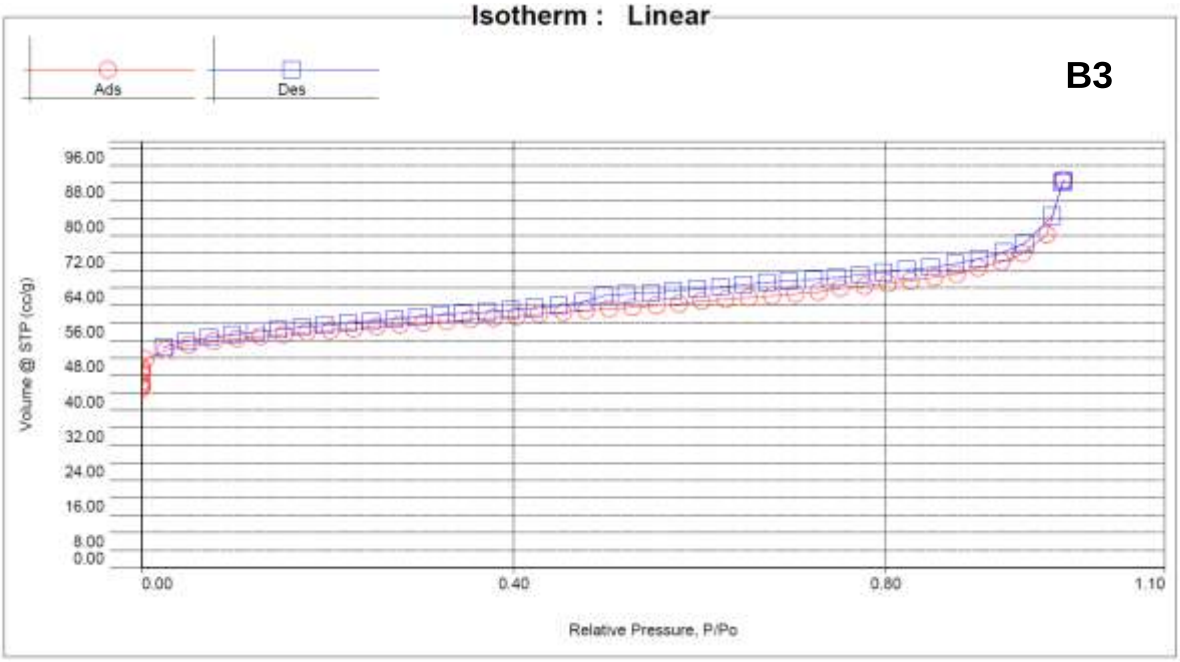

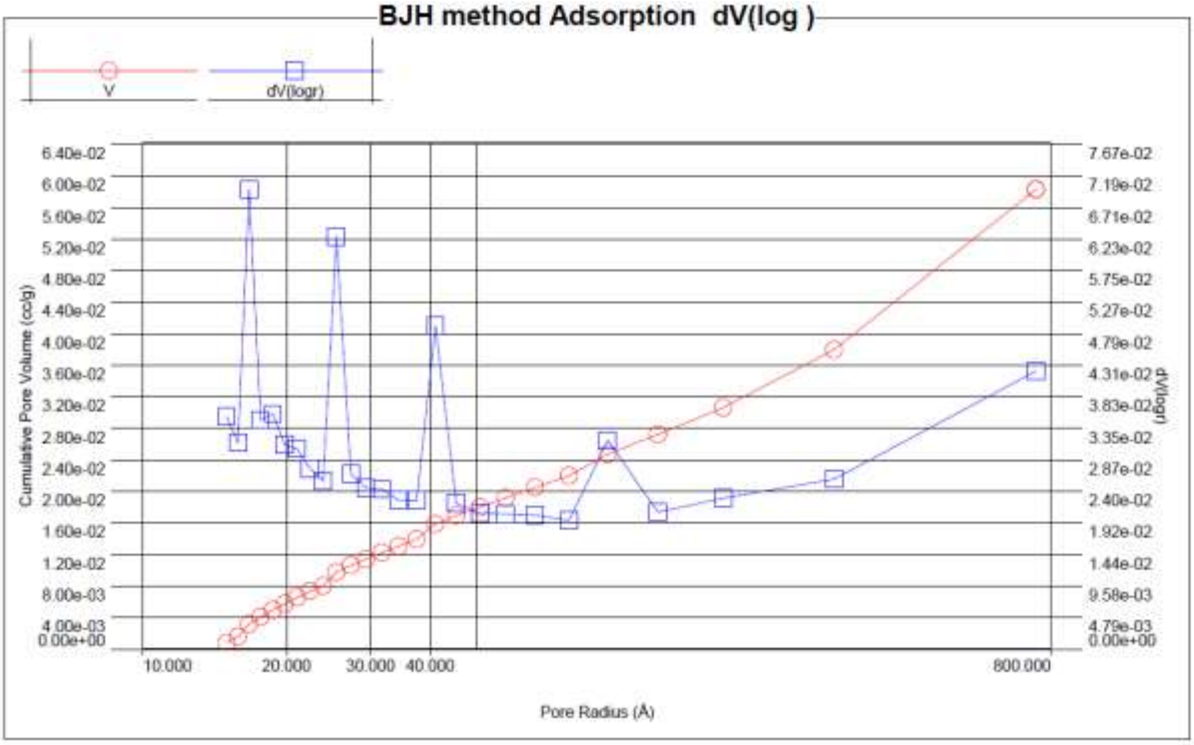

V-t method summary

Thickness method: DeBoer

**Slope =**	2.784
**Intercept =**	42.020
**Correlation coefficient, r =**	0.999335
**Micropore volume =**	0.065 cc/g
**Micropore area =**	168.523 m²/g
**External surface area =**	43.177 m²/g

Total Pore Volume data

Total Pore Volume

Total pore volume = 1.375e-01 cc/g for
pores smaller than 1118.9 Å (Radius)
at P/Po = 0.99134

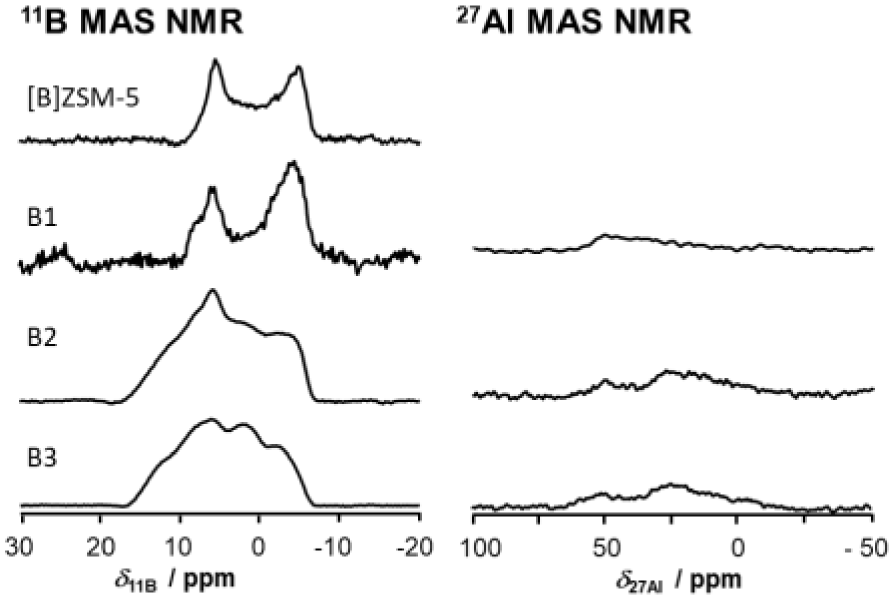

Figure S6: On the left ^{11}B MAS NMR spectra of (from top to bottom) a [B]ZSM-5 zeolite directly synthesized with boron incorporation, and boric acid modified samples B1, B2, and B3. On the right the respective ^{27}Al MAS NMR spectra of samples B1, B2, and B3. All spectra taken in the dehydrated state.

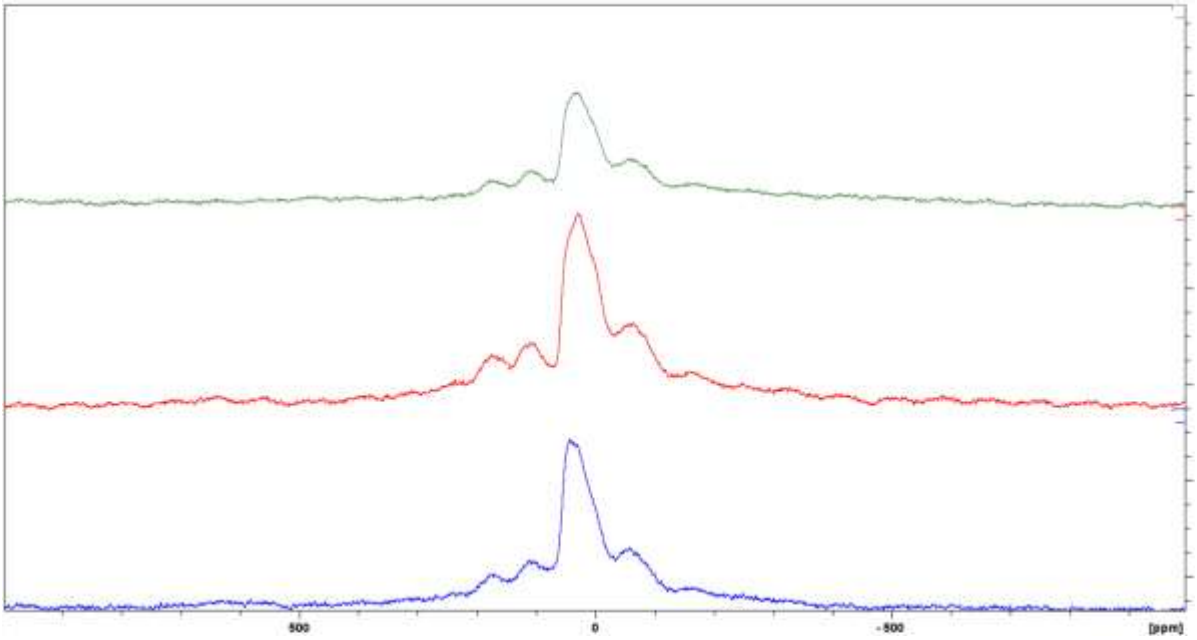

Figure S7: ^{27}Al MAS NMR spectra of Copper-modified Mordenites after formation of surface methoxy species. Unpublished data from[49].

Catalytic Results (Figures S8 to S15) for Ethanol and Ethene Conversion

ZSM-5 Si/Al = 20, WHSV = 4 h^{-1}

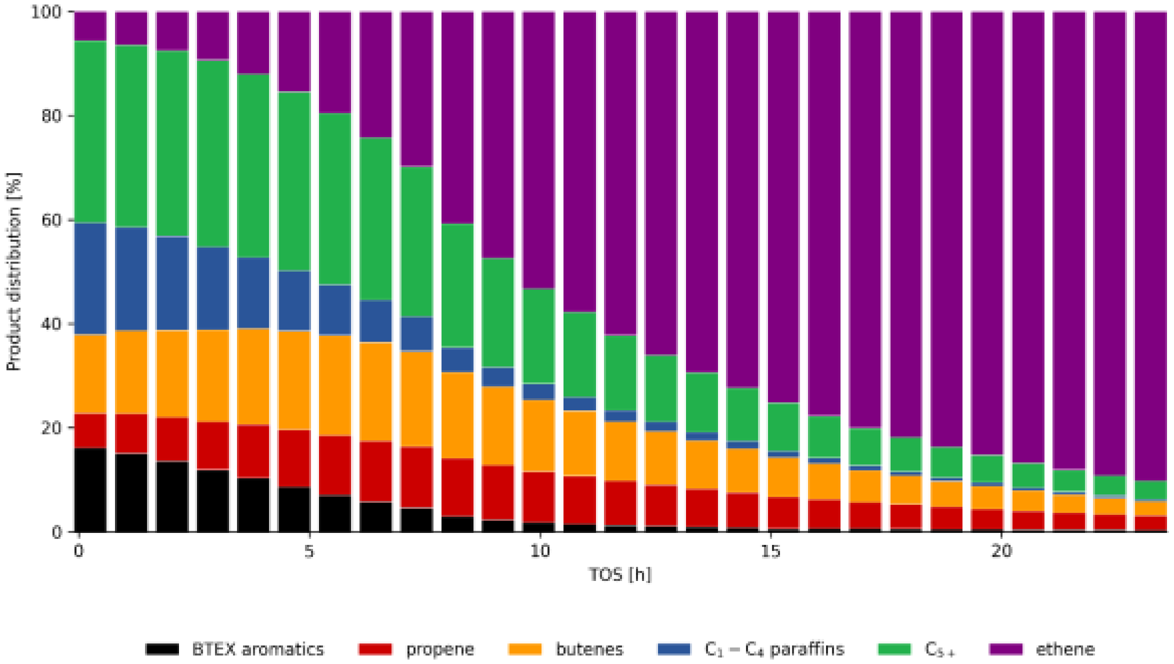

Feed: Ethanol (above), Ethene (below)

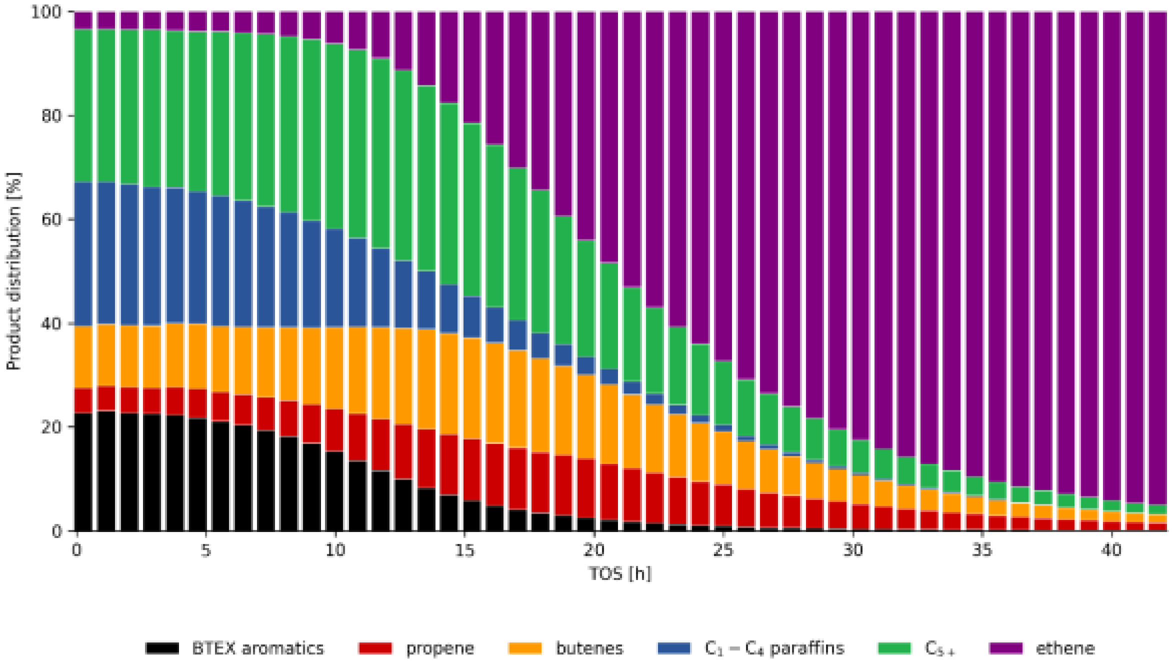

B1, WHSV = 4 h^{-1}

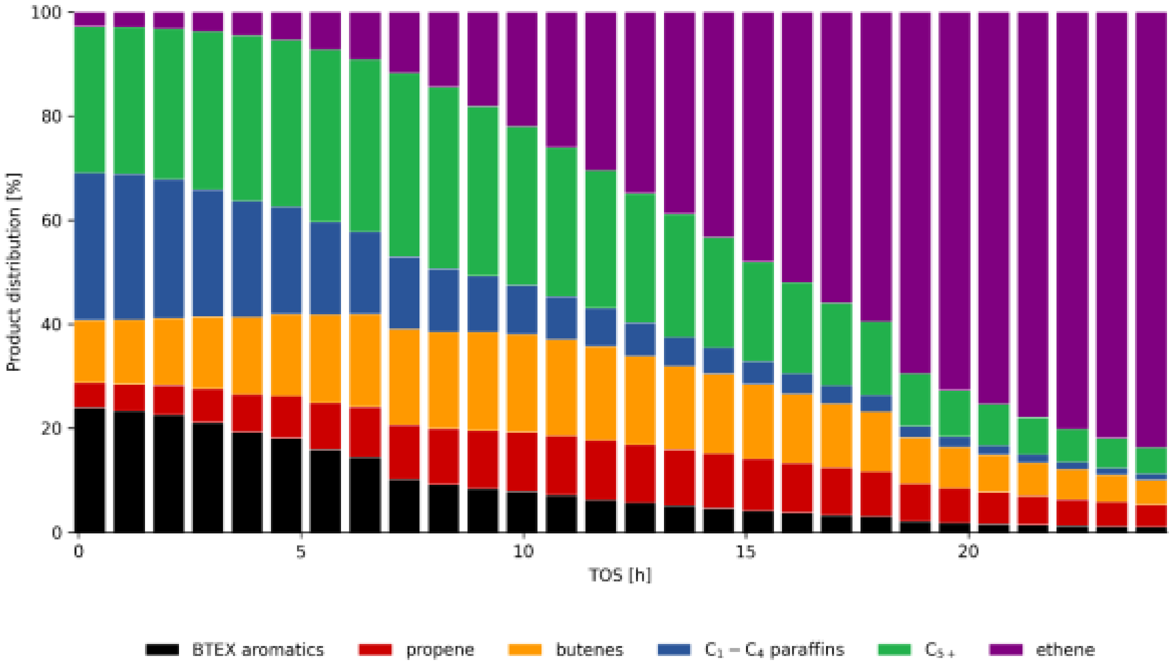

Feed: Ethanol (above), Ethene (below)

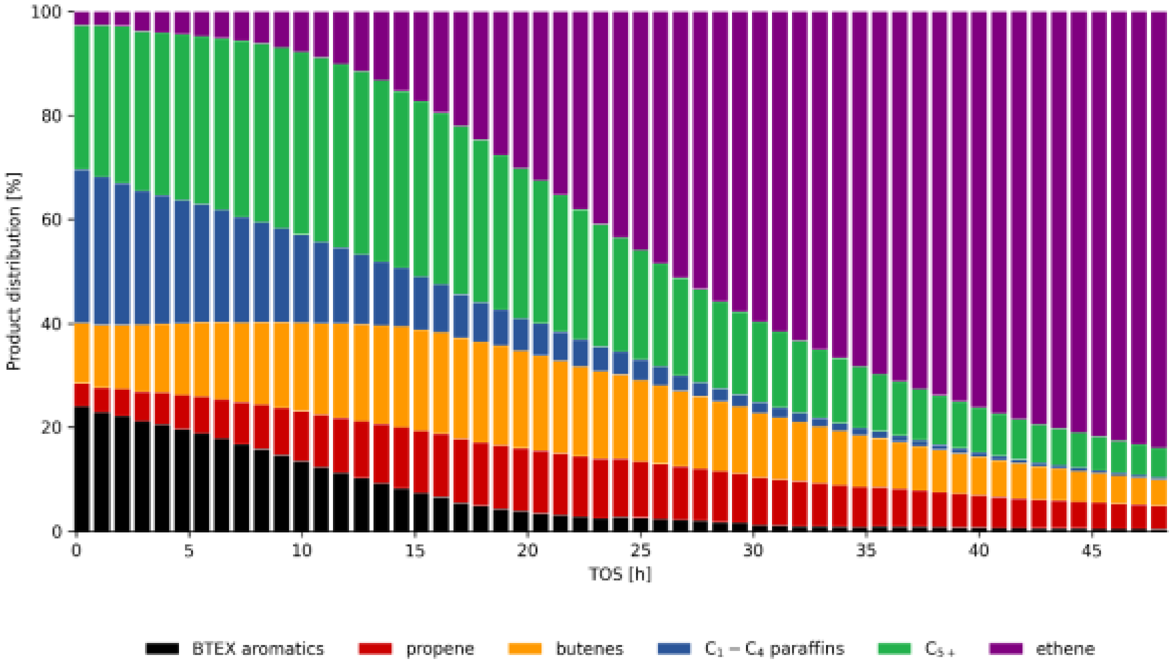

B2, WHSV = 4 h^{-1}

Mind: No trustworthy measurement for Ethanol possible due to blocked piping! Result originate from strongly reduced flow in piping. Conditions are thus not comparable to other measurements.

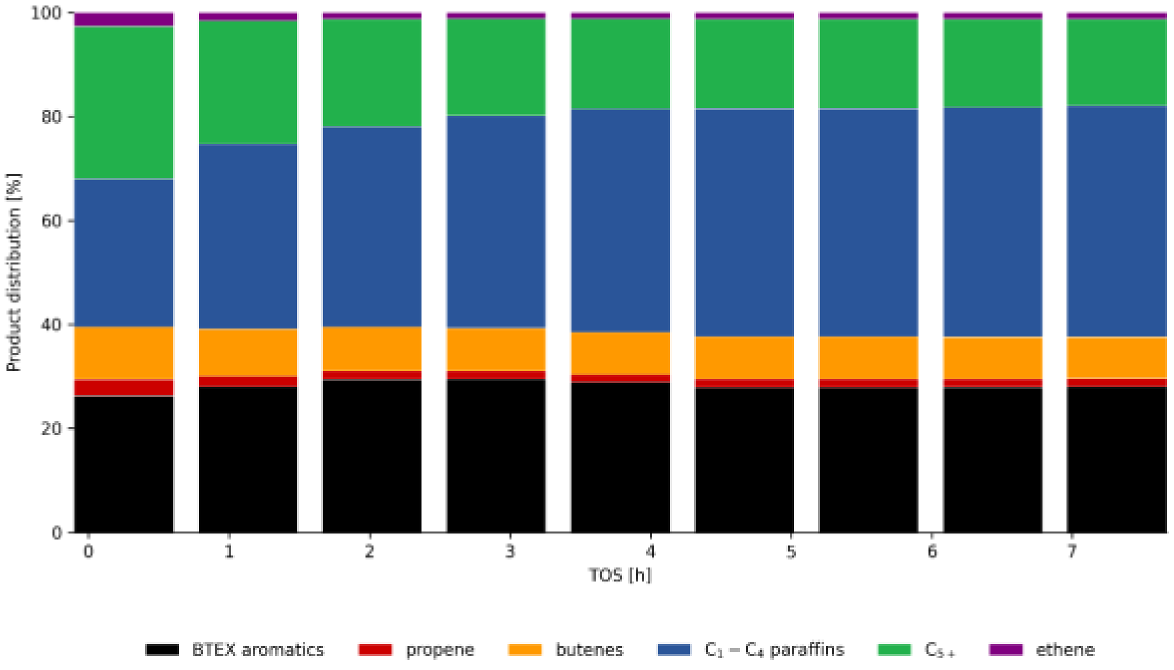

Feed: Ethanol (above), Ethene (below)

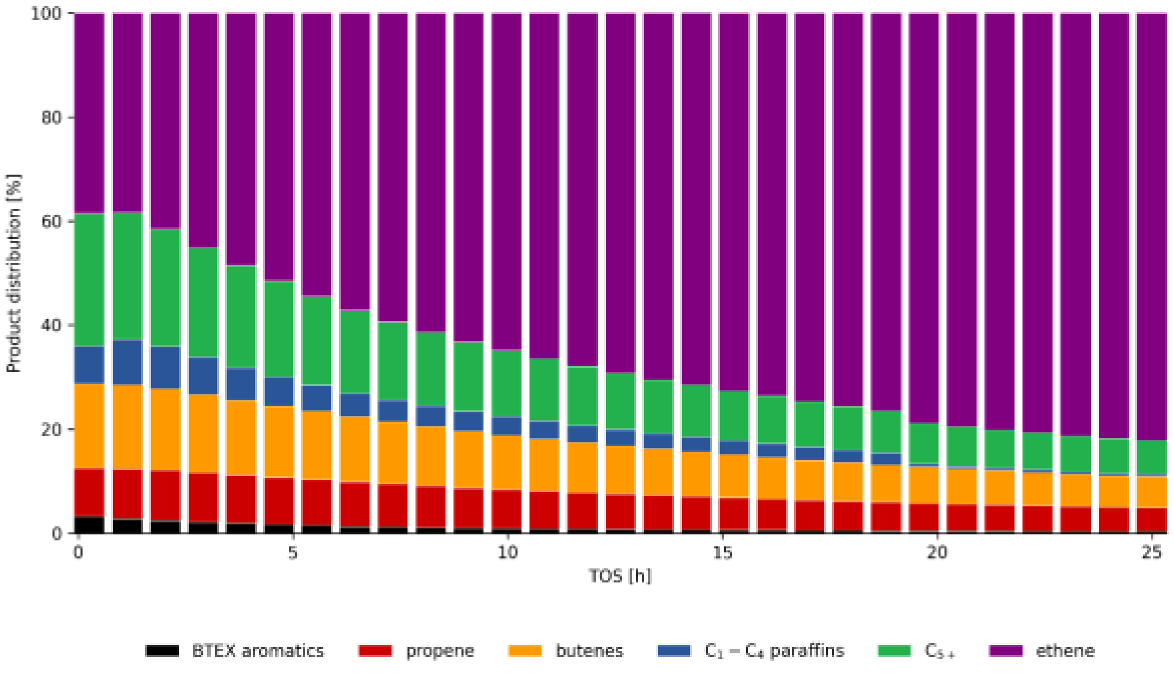

B3, WHSV = 4 h^{-1}

Mind: No trustworthy measurement for Ethanol possible due to blocked piping! Result originate from strongly reduced flow in piping. Conditions are thus not comparable to other measurements.

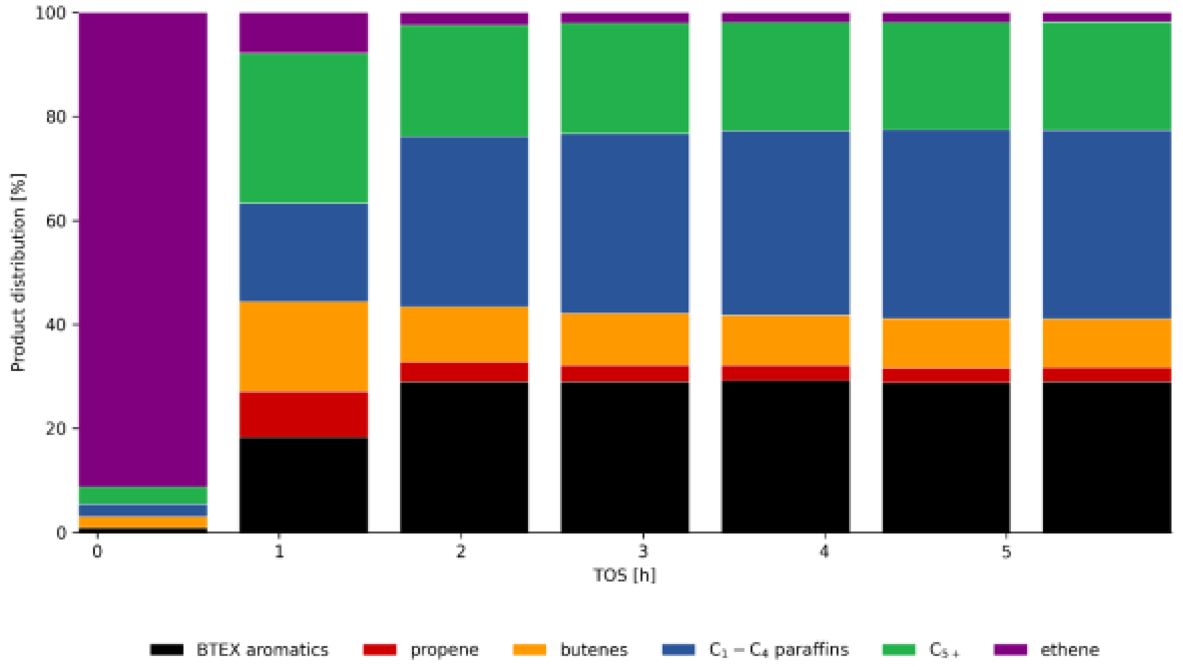

Feed: Ethanol (above), Ethene (below)

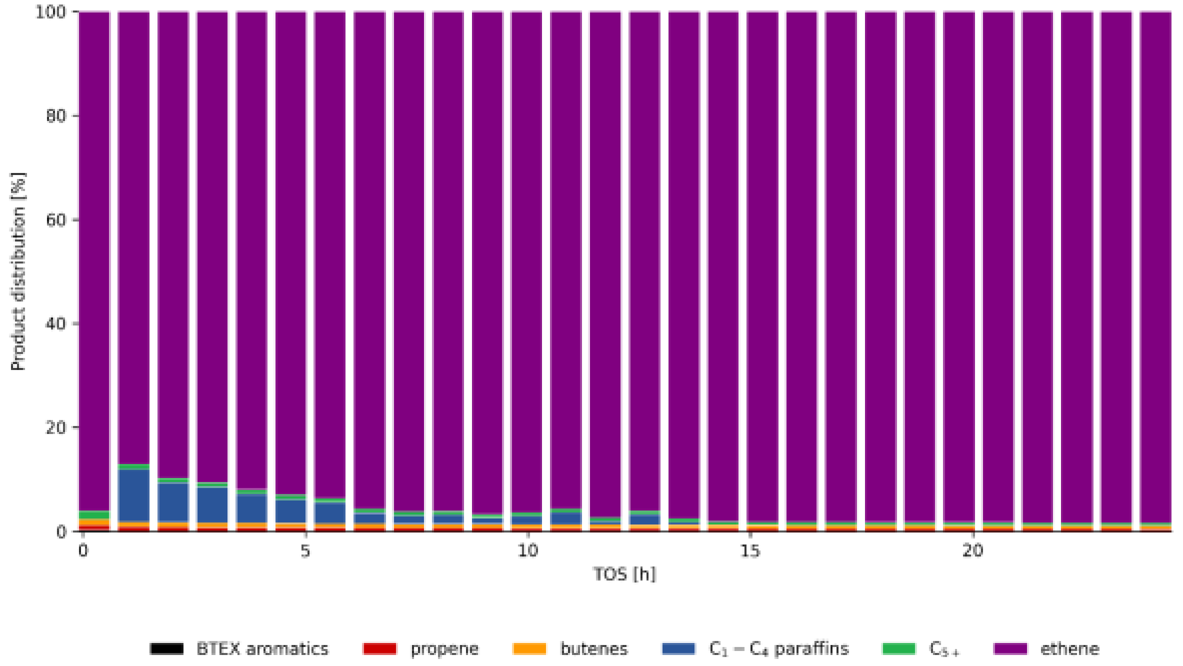

Table S1: Boron content after deactivation (with respect to mass of dry zeolite catalyst only).

	initial $B(OH)_3$ content [wt%]	after ethanol conversion deactivated, $B(OH)_3$ content [wt%]	after ethene conversion deactivated, $B(OH)_3$ content [wt%]
[Al]ZSM-5	0	0	0
B1	1.5	0.2	0.1
B2	5.4	0	0.7
B3	10.4	0.2	1.4

Catalytic Results for Methanol-to-Olefin (MTO) Conversion

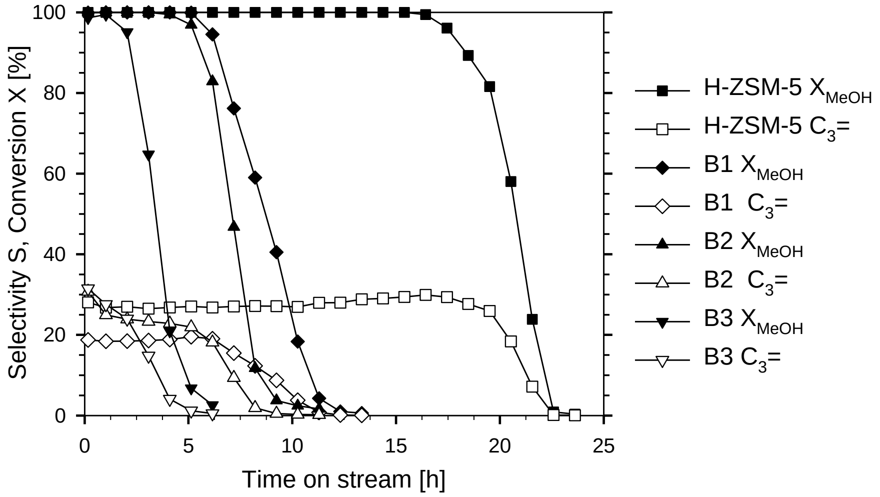

Figure S16: MTO conversion (X_{MeOH}) and propylene content ($C_{3=}$) over ZSM-5 zeolite for the parent H-ZSM-5 and after boric acid modification (B1, B2, B3) at WHSV = 4.0 h^{-1} and T = 723 K.

As in ETA conversion, the parent outperforms the boric acid modified catalysts in the MTO conversion reaction. All catalysts show an initial 100% conversion, but this value drops fast upon testing boric acid modified zeolites. The lifetime of the catalysts is decreasing with increasing boric acid loading in the order [Al]ZSM-5>>B1>B2>B3 and thereby this key parameter is reduced by up to 80%. The coke content of the samples likewise decreases in the same order, being 12% for the parent [Al]ZSM-5, 10.9% for B1, 10.7% for B2, and 7.7% for B3. It is remarked, that the coke accumulates much faster on boric acid modified samples. Thus the decreased lifetime by the additional boric acid species in the pores that hinder produced reactants from leaving the pores.
